# Supplementary material for: Natural transformation allows transfer of SCCmec-mediated methicillin resistance in Staphylococcus aureus biofilms
Source: Nat Commun. 2022 May 5;13:2477. doi: 10.1038/s41467-022-29877-2 (PMC9072672; doi:10.1038/s41467-022-29877-2)
Supplement: Supplementary file 1 — Supplementary Information file [file 41467_2022_29877_MOESM1_ESM.pdf]

Supplementary Information

Natural transformation allows transfer of SCC*mec*-mediated methicillin resistance in *Staphylococcus aureus* biofilms

Mais Maree, Le Thuy Thi Nguyen, Ryosuke L. Ohniwa, Masato Higashide, Tarek Msadek, Kazuya Morikawa

This file includes:

Supplementary Figures 1-20

Supplementary Tables 1-7

Supplementary References

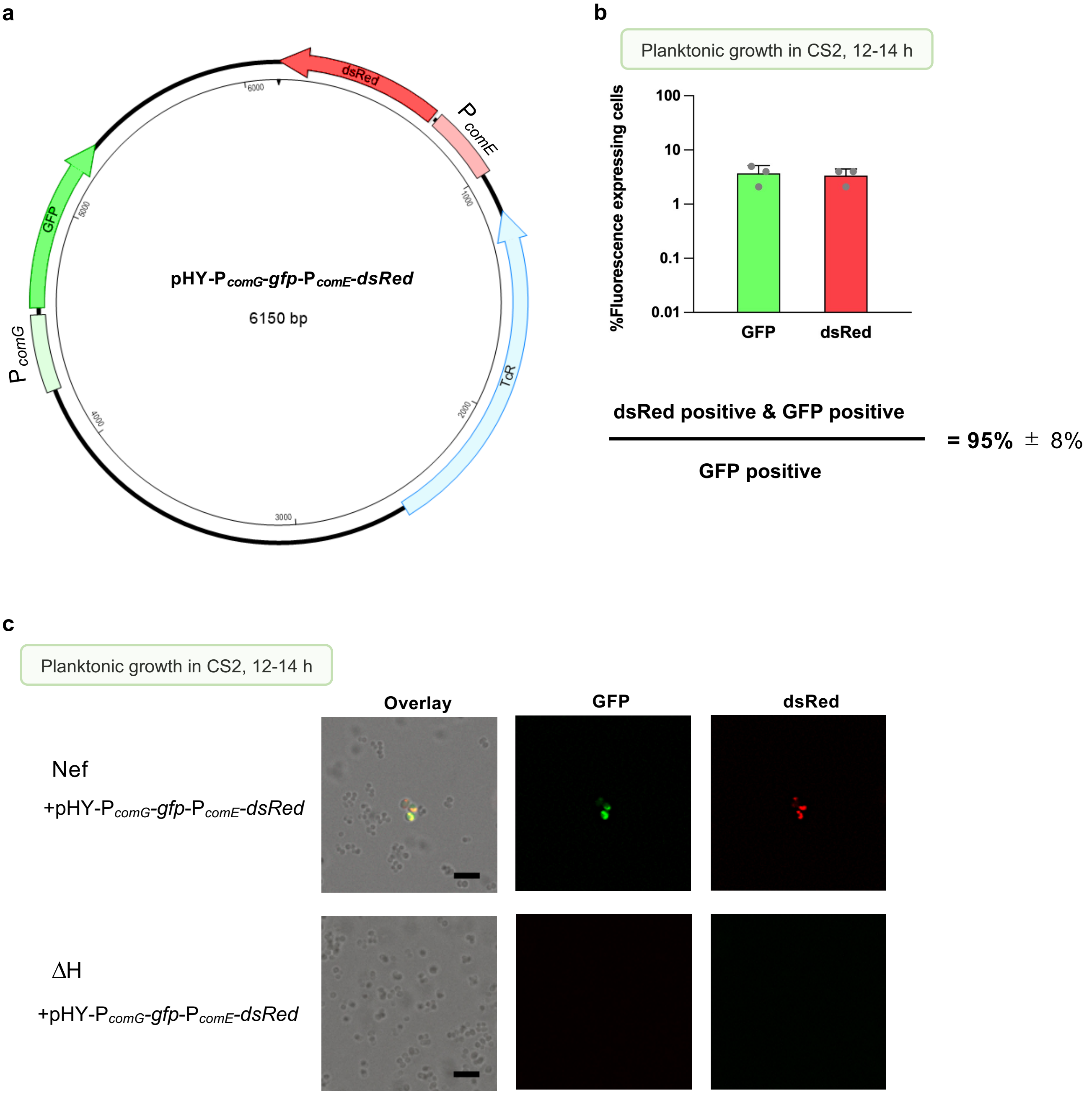

Supplementary Figure 1. P$_{comG}$ activity is a proper indicator of competence genes expression.
a, Schematic map of the dual reporter plasmid pHY-P$_{comG}$-*gfp*-P$_{comE}$-*dsRed.*
b, Nef carrying pHY-P$_{comG}$-*gfp*-P$_{comE}$-*dsRed* was grown in CS2 medium with shaking. The population percentage expressing the reporter was determined after 12-14 h of growth by fluorescent microscopy. At least 100 cells were counted in each independent experiment. The mean of n = 3 independent experiments is shown with SD. 95% of GFP positive cells were also positive in dsRed, indicating that P$_{comG}$ activity is a suitable indicator for competence gene expression.
c, Fluorescence microscopy images of Nef and ΔH carrying pHY-P$_{comG}$-*gfp*-P$_{comE}$-*dsRed*. No signals were detected from ΔH carrying pHY-P$_{comG}$-*gfp*-P$_{comE}$-*dsRed*. Scale bars, 5 μm.
The experiment was repeated at least three times independently with similar results. Source data are provided as a Source Data file.

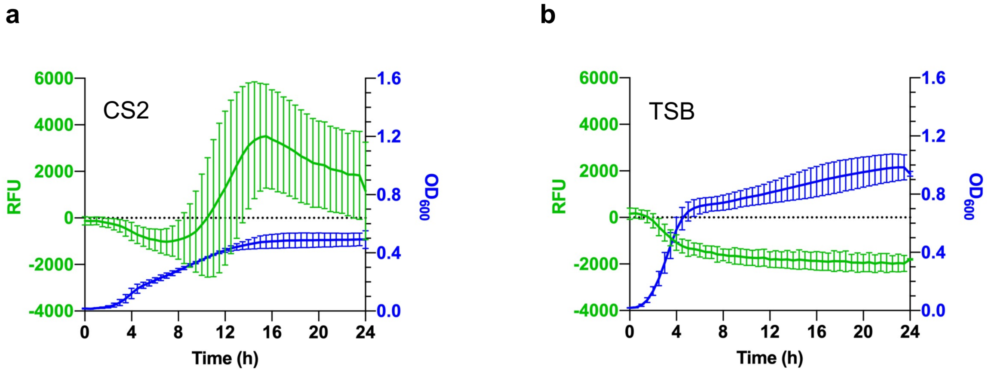

Supplementary Figure 2. Reporter assay for *comG* promoter activity.
Nef carrying the P_{comG}-*gfp* reporter was grown in either CS2 medium (**a**) or TSB (**b**) with shaking. Fluorescence intensity (green line) and OD_{600} (blue line) were measured every 30 min. The mean of n = 3 independent experiments is shown with SD. Source data are provided as a Source Data file.

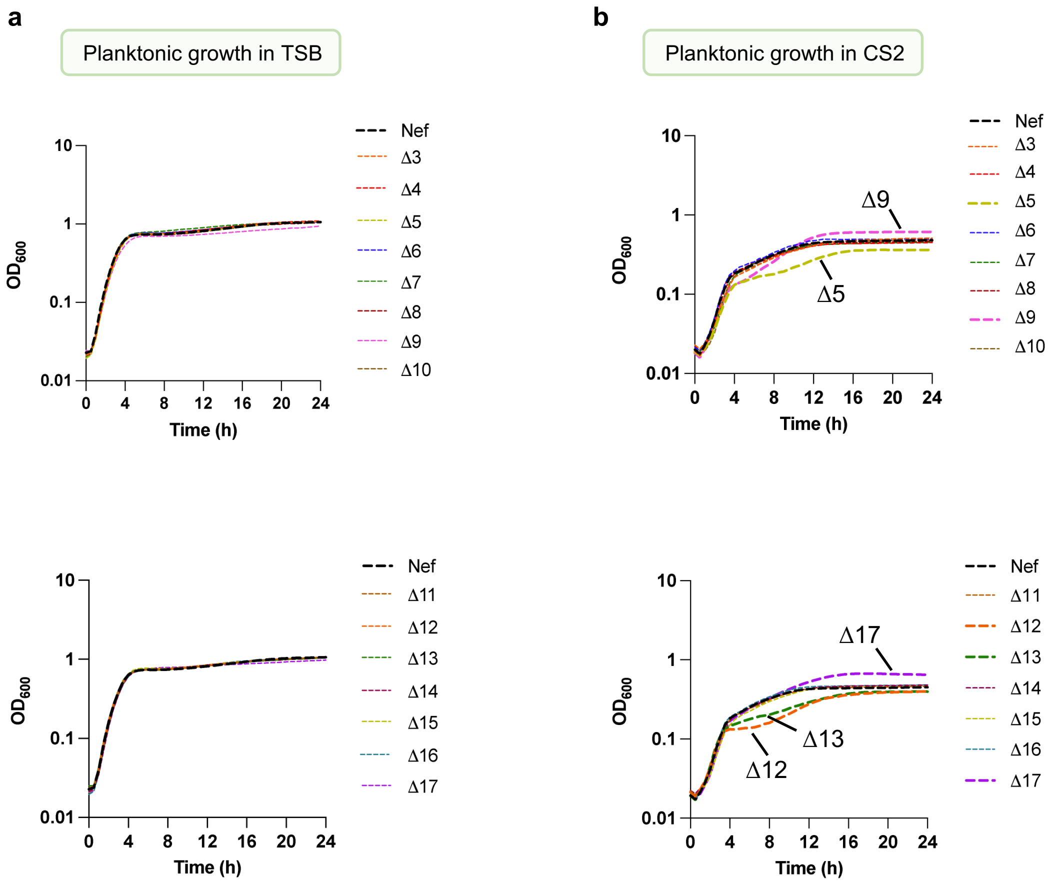

Supplementary Figure 3. Growth curves of Nef and its derivative ΔTCS.
Cells were grown in either TSB (**a**) or CS2 medium (**b**) with shaking. OD_{600} was measured every 30 min. The mean of n = 3 independent experiments is shown. Error bars are omitted for clarity, but all standard deviations did not exceed ± 0.144. Source data are provided as a Source Data file.

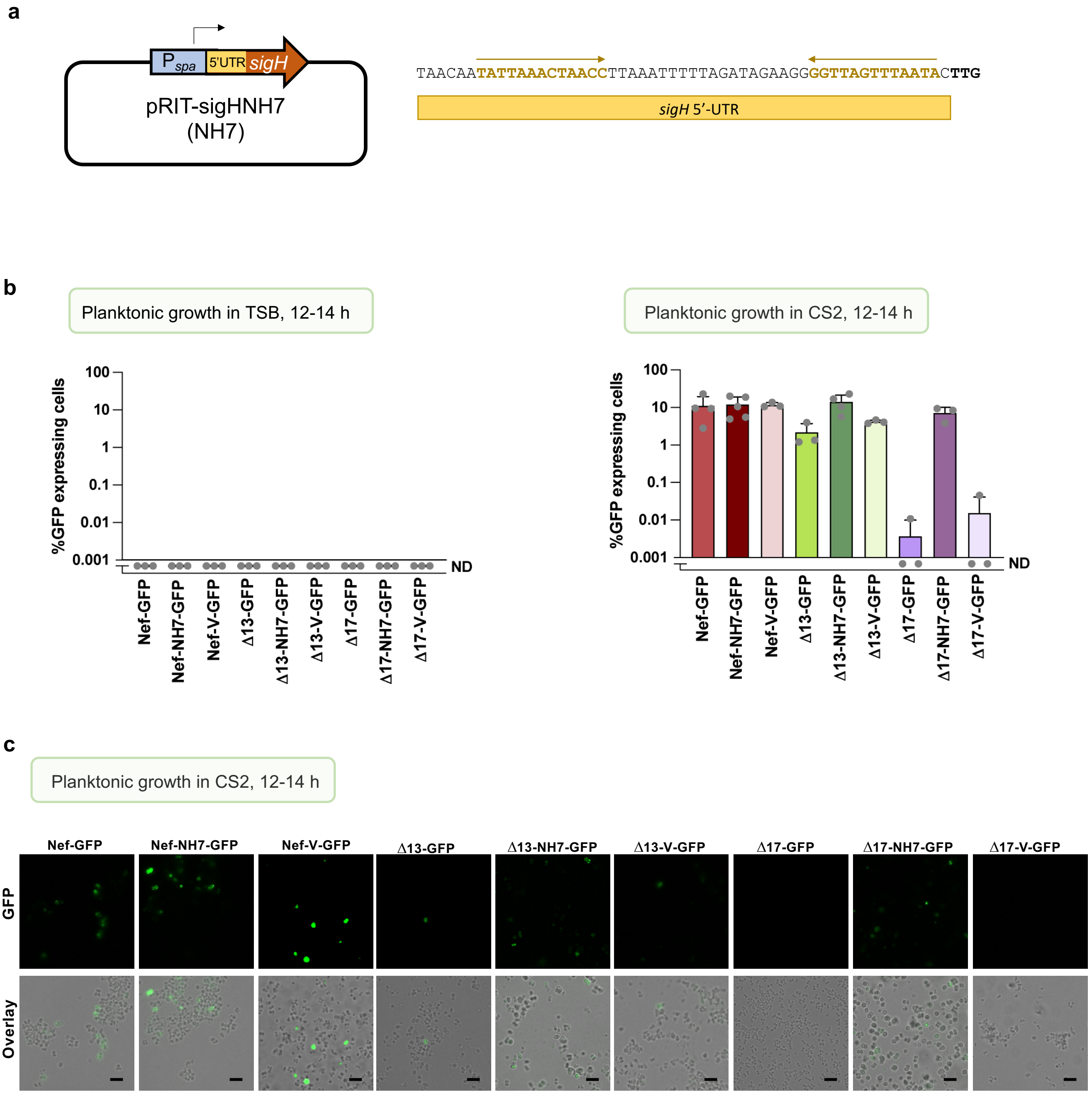

Supplementary Figure 4. Absence of TCS13 and TCS17 can be compensated for by *sigH* expression in P_{comG} expression.

a, Schematic map of pRIT-sigHNH7 plasmid (NH7). The *sigH* mRNA including its 5'-UTR region is constitutively expressed under the *spa* promoter (P_{spa}). The *sigH* 5'-UTR includes inverted repeat sequences that suppress SigH translation.

b, The population percentage of Nef and its derivatives expressing P_{comG}-*gfp* reporter with or without NH7 plasmid was determined after 12-14 h of growth by fluorescent microscopy. The vector control strains are shown by "-V-" within the strain name, e.g., Nef-V-GFP. Cells were grown in TSB or CS2 medium with shaking. At least 200 cells were counted in each experiment. The mean of at least n = 3 independent experiments is shown with SD. ND: none detected.

c, Fluorescence microscopy images of the cells grown in CS2 for 12-14 h. Scale bars, 5 μm.

The experiment was repeated at least three times independently with similar results. Source data are provided as a Source Data file.

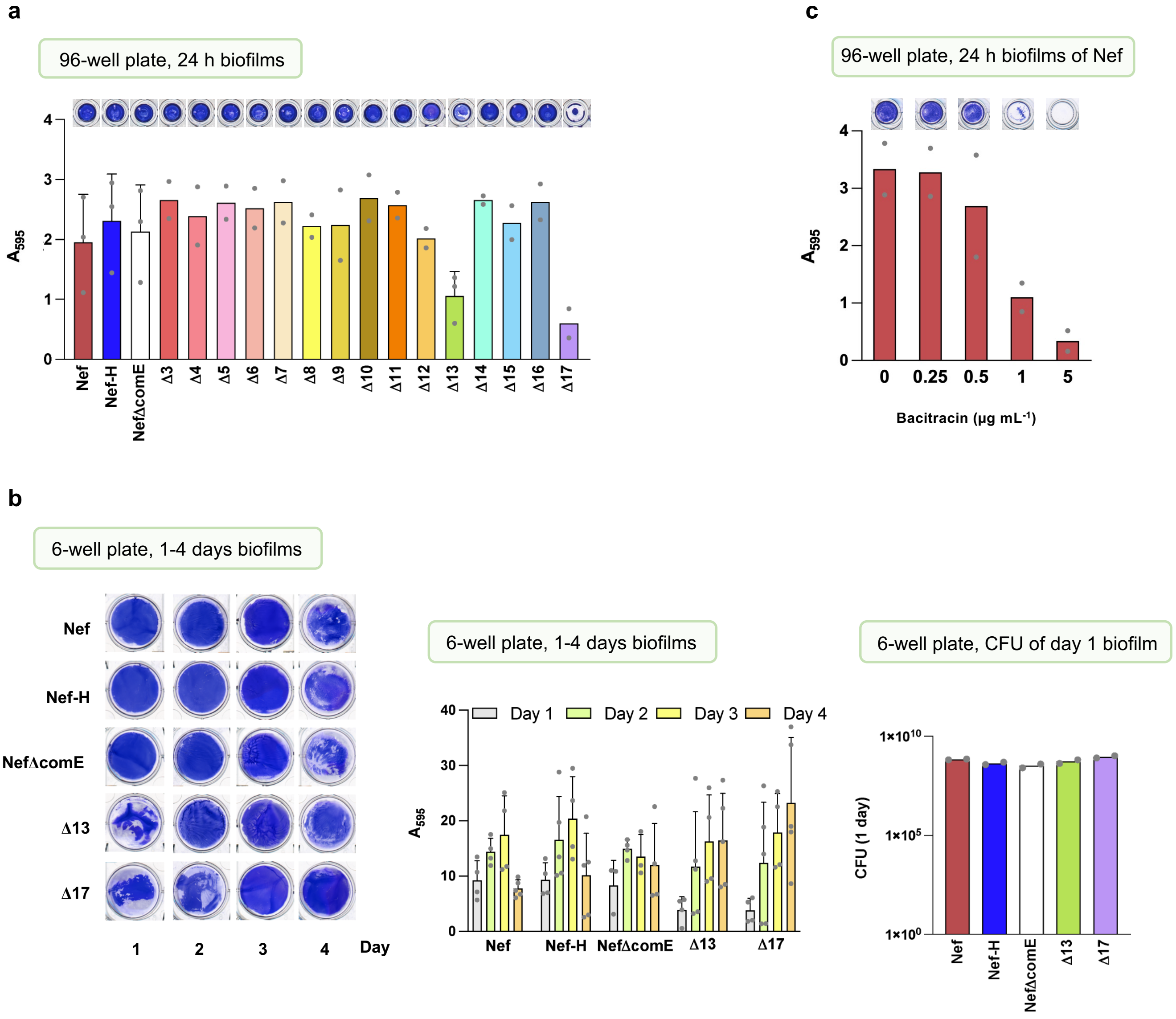

Supplementary Figure 5. Biofilm formation is impaired in the TCS13 and TCS17 mutants.
a, Nef and its derivatives were statically grown in CS2 medium in a 96-well plate for 24 h. The biofilm was quantified as described in Methods. The mean of at least n = 2 independent experiments is shown. Error bars represent SD. **b,** left and middle, Time-course of biofilm quantity in Nef and its derivatives. The cells were statically grown in CS2 medium in a 6-well plate for up to 4 days. The mean of at least n = 3 independent experiments is shown with SD.
b, right. CFU of the whole cells after 1 day (without washing non-biofilm cells).
c, Low concentration of bacitracin prohibits Nef biofilm formation in CS2 medium. The mean of n = 2 independent experiments is shown. Source data are provided as a Source Data file.

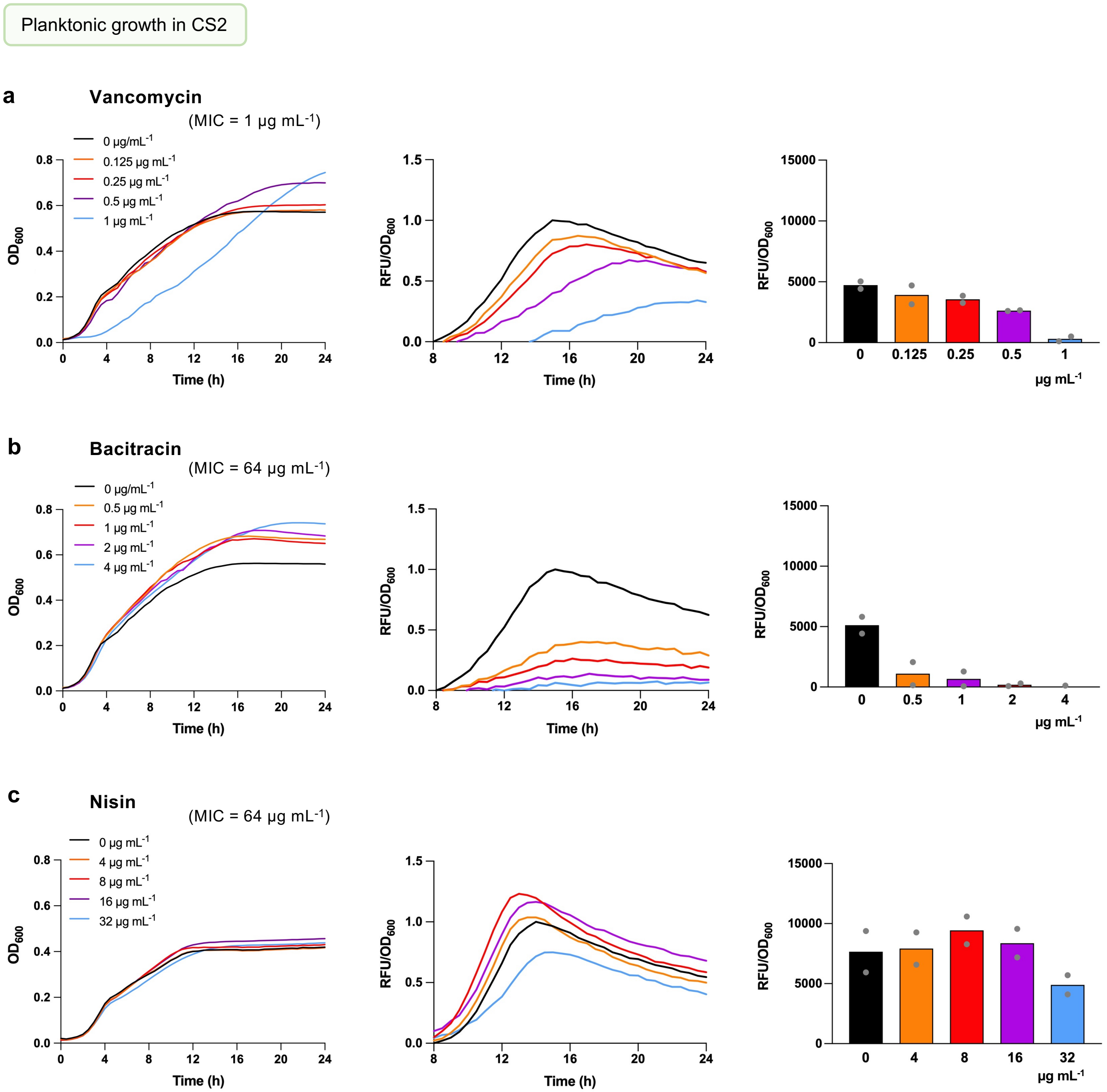

Supplementary Figure 6. *comG* promoter activity is affected by cell wall-targeting antibiotics. Nef-GFP was grown with vancomycin (**a**), bacitracin (**b**), or nisin (**c**) in CS2 medium with shaking for 24 h. Fluorescence (RFU) and OD_{600} were measured every 30 min. Data shown are OD_{600} values (**left panels)**, RFU/OD_{600} values **middle panels**) or increases in RFU/OD_{600} values during 8-24 h of growth (**right panels**). The mean of n = 2 independent experiments is shown. Source data are provided as a Source Data file.

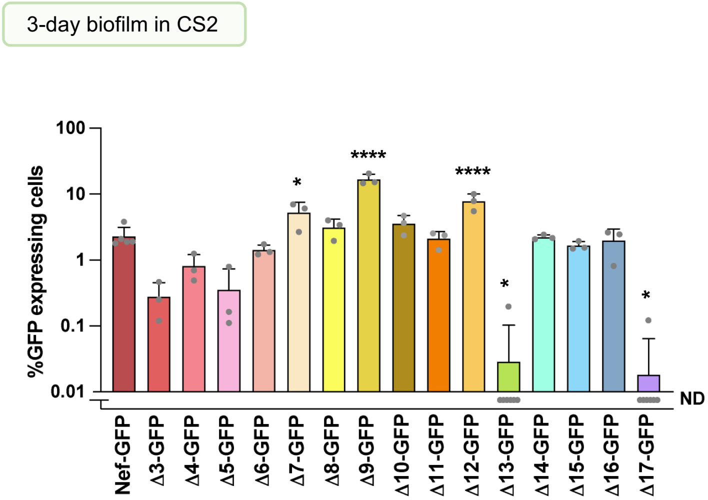

Supplementary Figure 7. *comG* promoter activity is affected by multiple TCSs in biofilm.

Nef and its derivative ΔTCSs carrying the P_{comG}-*gfp* reporter were statically grown in CS2 medium for 3 days. The percentage of GFP-expressing cells was calculated as observed by fluorescence microscopy. Bars represent the mean of at least n = 3 independent experiments with error bars indicating SD. ND: none detected. Statistical significance was determined by one-way ANOVA with Tukey's multiple comparison test. Nef-GFP vs Δ7-GFP $^{*}P = 0.0107$, Nef-GFP vs Δ13-GFP $^{*}P = 0.0159$, Nef-GFP vs Δ17-GFP $^{*}P = 0.0152$, $^{****}P<0.0001$. Source data are provided as a Source Data file.

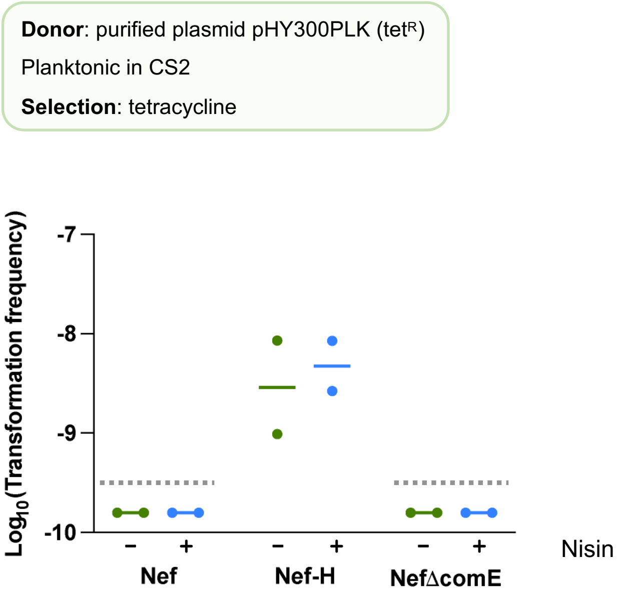

Supplementary Figure 8. Nisin has no detectable effect on natural transformation.

Nef and its derivatives were grown in CS2 medium with or without nisin (8 μg mL^{-1}). Transformation frequencies were determined after 10 h of planktonic growth. The transformants were selected by tetracycline. The dotted line represents the detection limit. The mean of n = 2 independent experiments is shown. Transformation remained undetectable, regardless of the nisin treatment, and there were no significant effects in the SigH-overexpressing strain (Nef-H). Source data are provided as a Source Data file.

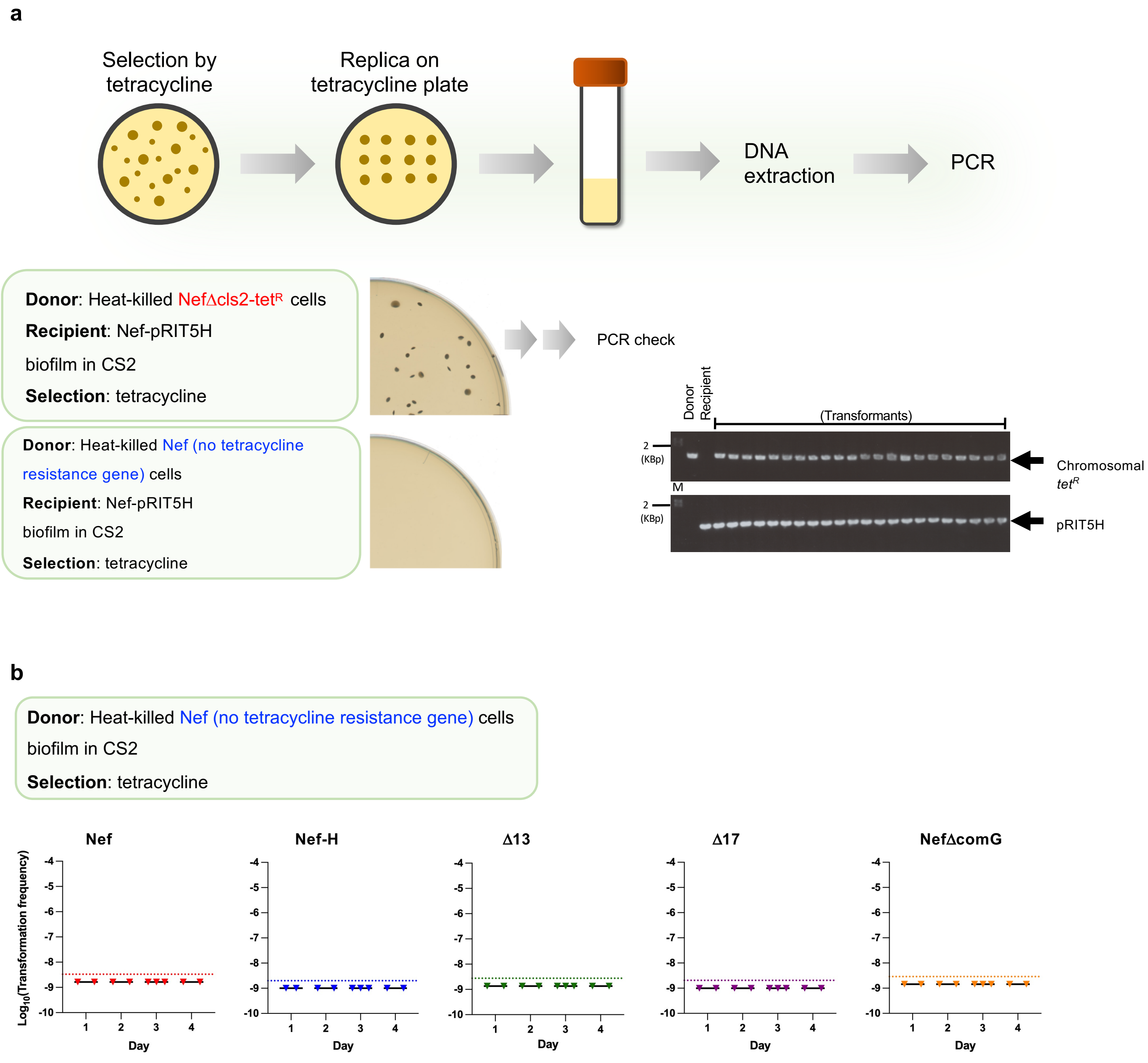

Supplementary Figure 9. Spontaneous tetracycline resistant colonies do not emerge.
a, Transformants were selected by tetracycline. Emergent colonies were replicated on fresh tetracycline plates. These replicate colonies were then inoculated in liquid cultures followed by DNA extraction for PCR. A representative image of the colonies following the initial tetracycline selection is shown. In the absence of tetracycline resistance gene in donor (Nef), no colony emerges. The tetracycline resistance gene can be detected by PCR in the transformants as well as in the donor (NefΔcls2-tet^R). The pRIT5H plasmid in the recipient (Nef-pRIT5H) was detected in the transformants. M: DNA size marker, λ HindIII.
b, When a heat-killed Nef donor (with no tetracycline resistance gene) is used, no colonies emerge in any recipient. Nef and its derivatives were statically grown in CS2 medium. Transformation frequencies were determined every 24 h. Dotted lines represent the detection limit. Data points represent independent experiments. Source data are provided as a Source Data file.

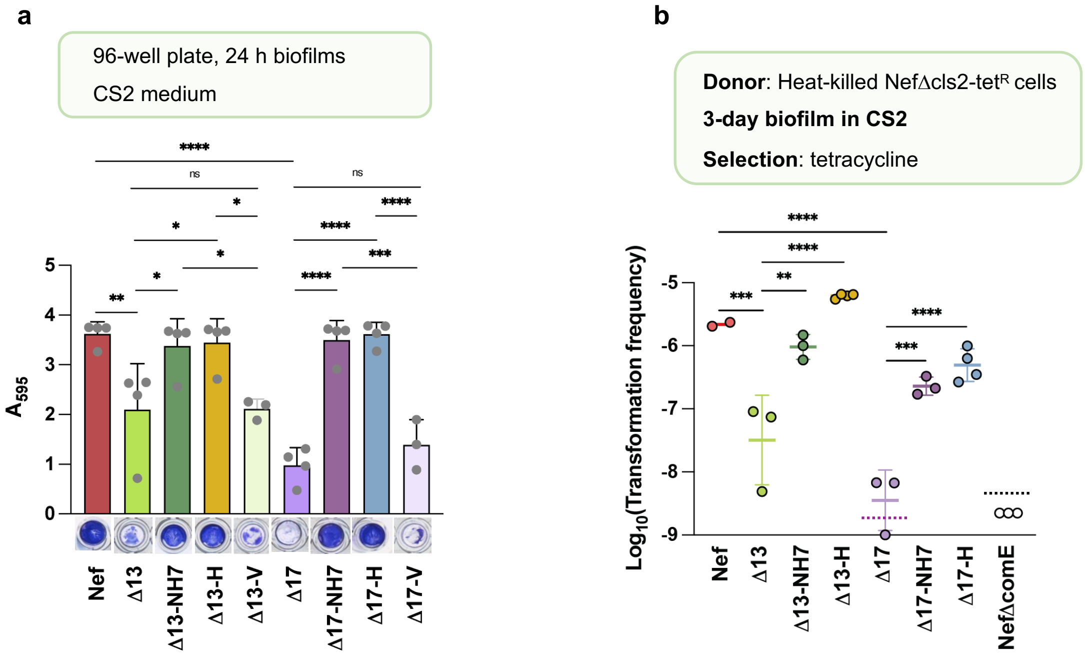

Supplementary Figure 10. Absence of TCS13 and 17 can be compensated for by expression of *sigH* in biofilm formation and transformation.

a, Quantification of 24 h biofilms of Nef and its derivatives. The *sigH* was overexpressed by pRIT-sigHNH7 (shown by "-NH7" in strain names) or SigH by pRIT-sigH ("-H"): see Supplementary Figure 4 regarding these plasmids. Vector controls are shown by "-V". The mean of at least n = 3 independent experiments is shown with SD. The *sigH*/SigH overexpression restored the biofilm formation in both Δ13 and Δ17, while vector control did not. Statistical significance was determined by one-way ANOVA with Tukey's multiple comparison test. Nef vs. Δ13 **P=0.0044, Δ13 vs. Δ13-NH7 *P=0.0243, Δ13 vs. Δ13-H *P=0.0156, Δ13-V vs. Δ13NH7 *P=0.0497, Δ13-V vs. Δ13-H *P=0.0337, Δ17-V vs. Δ17-NH7 ***P=0.0002, ****P<0.0001, ns P>0.9.

b, Transformation frequencies of Nef and its derivatives were determined at day 3 in the biofilm growth condition. Heat-killed NefΔcls2-tet^{R} donor was used. Transformants were selected by tetracycline. The dotted lines represent the detection limit. The mean of at least n = 2 independent experiments is shown. Error bars represent SD. ND: None detected, $<10^{-9}$. The *sigH*/SigH overexpression restored the transformation frequencies in both Δ13 and Δ17. NefΔcomE served as a negative control for the transformation assay. Statistical significance was determined by one-way ANOVA with Tukey's multiple comparison test. Nef vs. Δ13 ***P=0.0002, Δ13 vs Δ13-NH7 ***P=0.0007, **** P < 0.0001.

Source data are provided as a Source Data file.

a

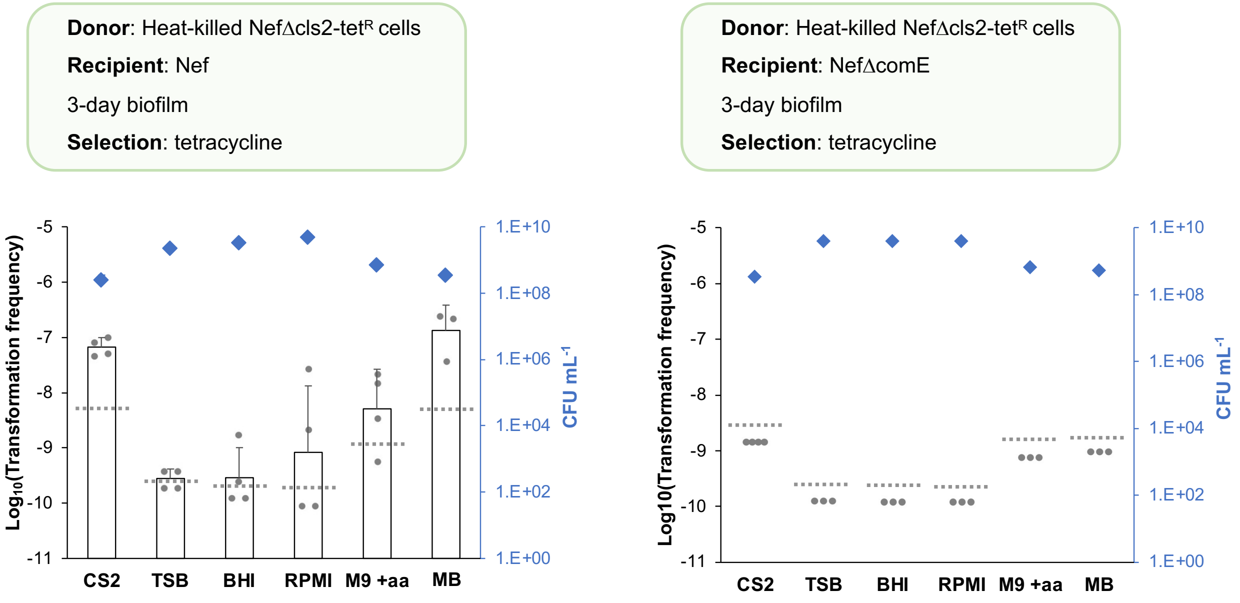

b

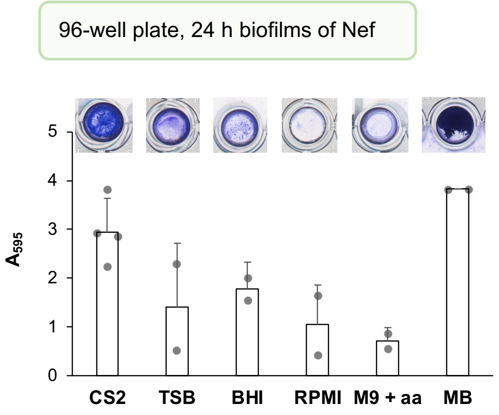

Supplementary Figure 11. Biofilm formation and natural genetic transformation in distinct growth media.

a, Nef (left) and NefΔcomE (right) were statically grown in different growth media for 3 days. Transformation frequencies (bars) and CFU mL^{-1} (blue) were determined after 3 days. A heat-killed NefΔcls2-tet^R donor was used. Transformants were selected by tetracycline. The dotted lines represent the detection limit. The mean of at least n = 3 independent experiments is shown with SD.

b, Biofilm formation of Nef was assessed in 96-well plates after static growth for 24 h in different growth media. The mean of at least n = 2 independent experiments is shown.

Source data are provided as a Source Data file.

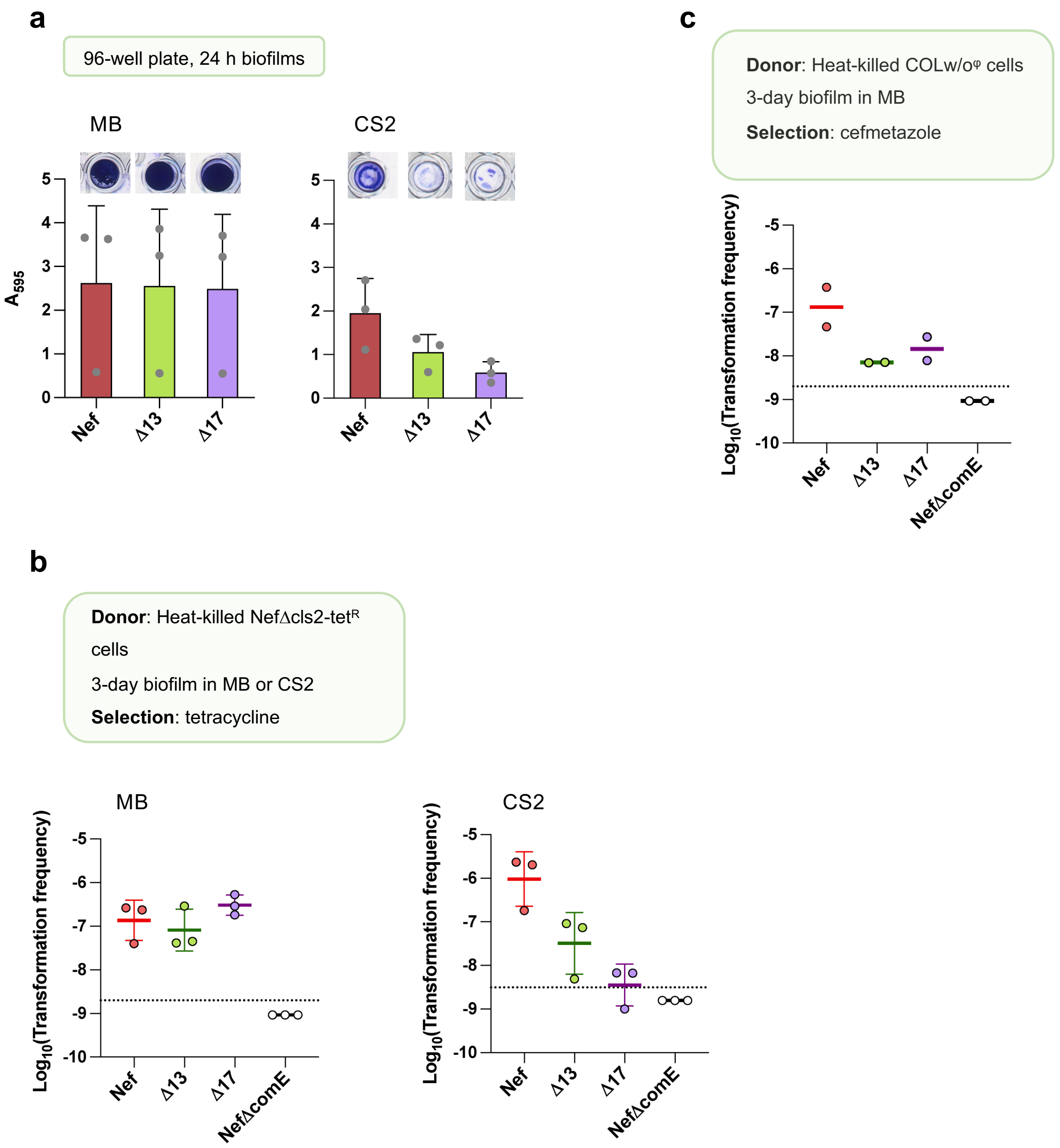

Supplementary Figure 12. TCS13 and TCS17 are dispensable for transformation in MB.
a-b, Cells were statically grown in MB (whole milk & BHI medium [1:1]) or CS2. **a,** Biofilm formation was assessed in 96-well plates after 24 h. The mean of n = 3 independent experiments is shown with SD.
b, Transformation frequencies were determined after 3 days. A heat-killed NefΔcls2-tet^R donor was used. Transformants were selected by tetracycline. Dotted lines represent the detection limit. The mean of n = 3 independent experiments is shown with SD.
c, Cells were statically grown in MB. Transformation frequencies were determined after 3 days. A heat-killed COLw/oφ donor was used. Transformants were selected by cefmetazole. Dotted lines represent the detection limit. The mean of n = 2 independent experiments is shown.
Source data are provided as a Source Data file.

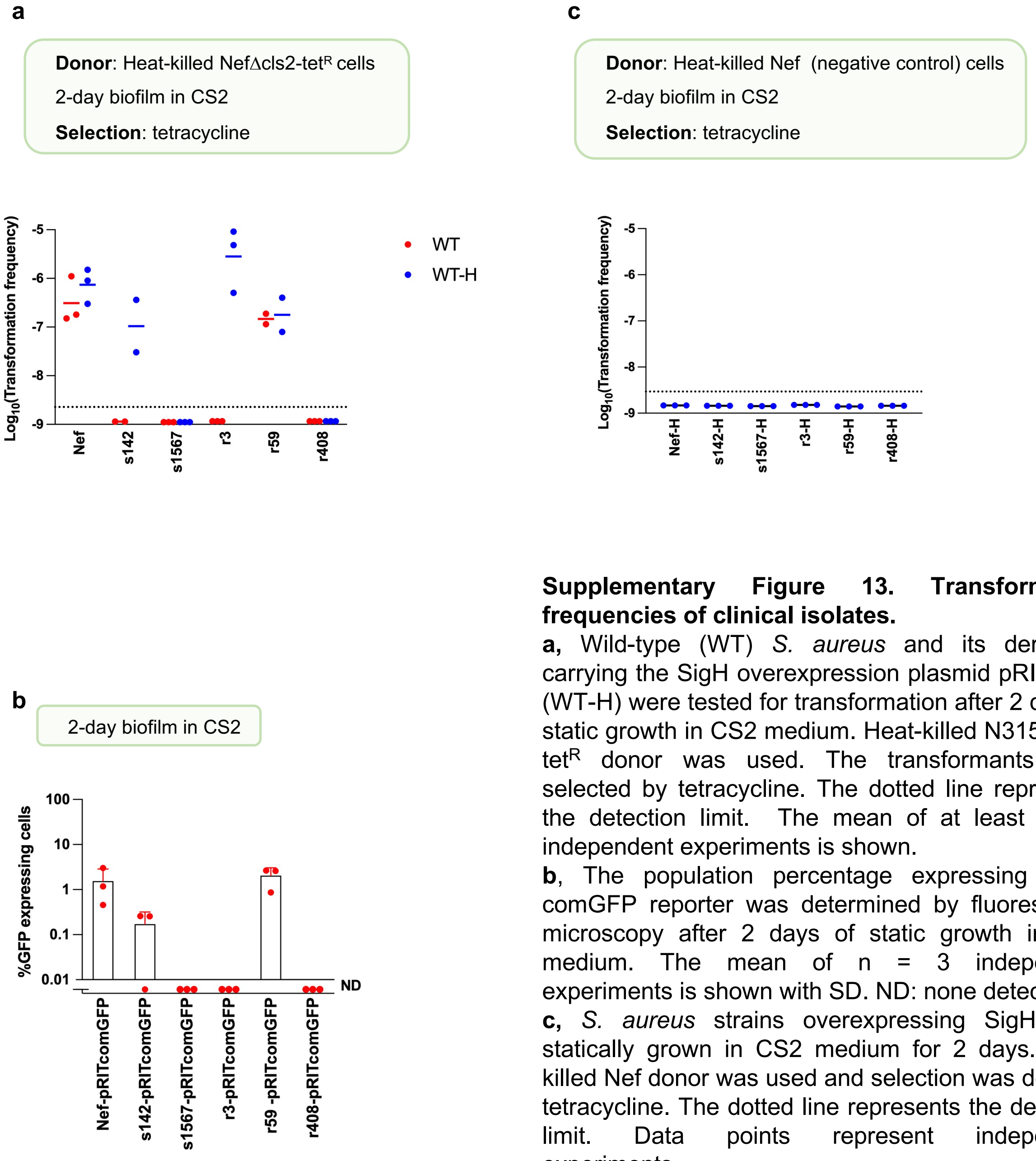

Supplementary Figure 13. Transformation frequencies of clinical isolates.

a, Wild-type (WT) *S. aureus* and its derivative carrying the SigH overexpression plasmid pRIT-sigH (WT-H) were tested for transformation after 2 days of static growth in CS2 medium. Heat-killed N315Δcls2-tet^R donor was used. The transformants were selected by tetracycline. The dotted line represents the detection limit. The mean of at least n = 2 independent experiments is shown.

b, The population percentage expressing pRIT-comGFP reporter was determined by fluorescence microscopy after 2 days of static growth in CS2 medium. The mean of n = 3 independent experiments is shown with SD. ND: none detected.

c, *S. aureus* strains overexpressing SigH were statically grown in CS2 medium for 2 days. Heat-killed Nef donor was used and selection was done by tetracycline. The dotted line represents the detection limit. Data points represent independent experiments.

Source data are provided as a Source Data file.

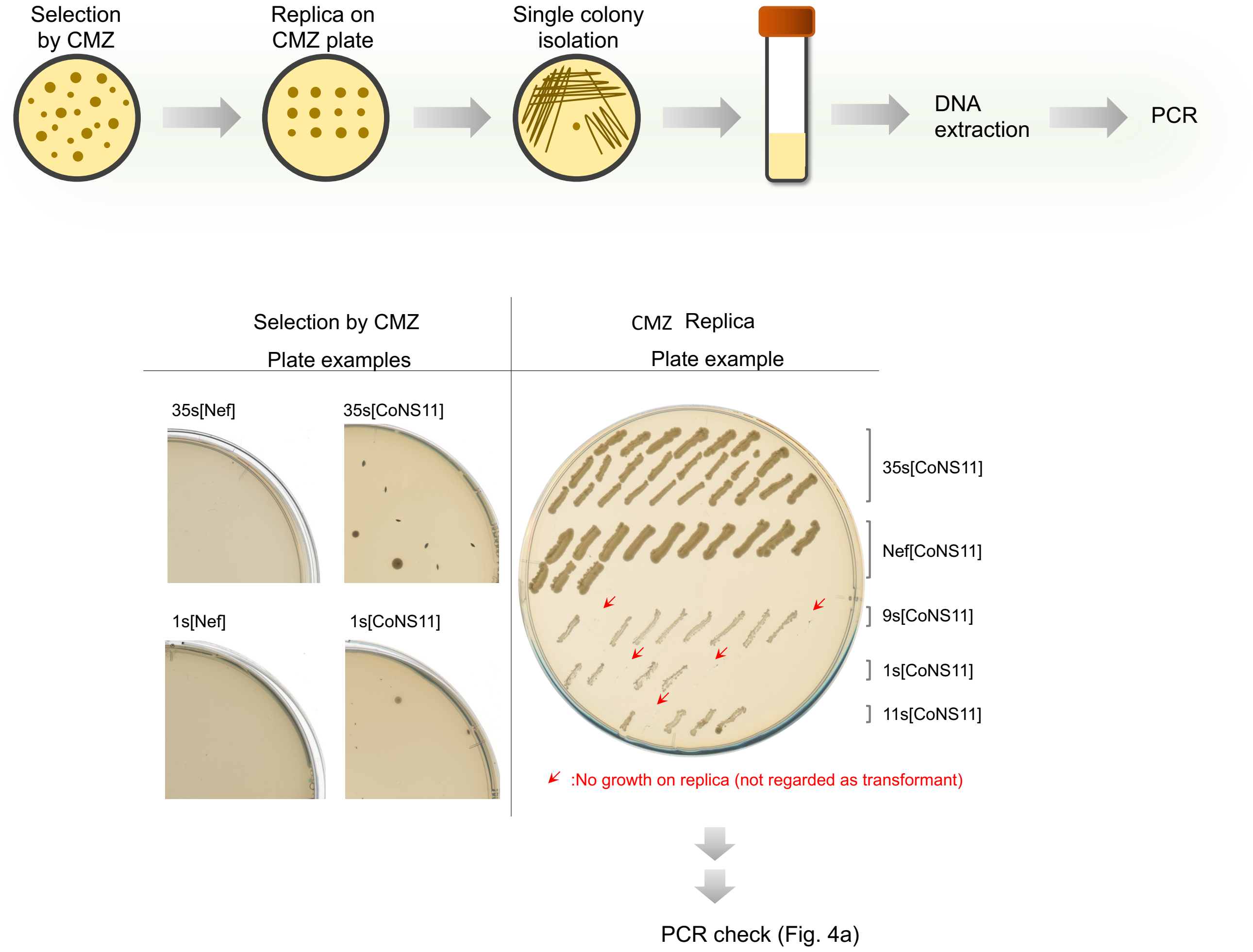

Supplementary Figure 14. The *mecA* transformant selection by cefmetazole (CMZ).
After transformation and selection by cefmetazole, generated colonies are replicated onto fresh agar plates containing cefmetazole. In order to prepare a PCR template, cells capable of growth on replica plates undergo single colony isolation and liquid culture to avoid any contamination of donor DNA. Images show representative plates following initial selection by cefmetazole (left) and replication (right). Tiny colonies tend not to grow on replica plates and are not counted as transformants (red arrows). Stable transformants make rigid colonies on replica plates (e.g. 35s[CoNS11]) but unstable ones also grow on replica plates (e.g. 9s[CoNS11]).

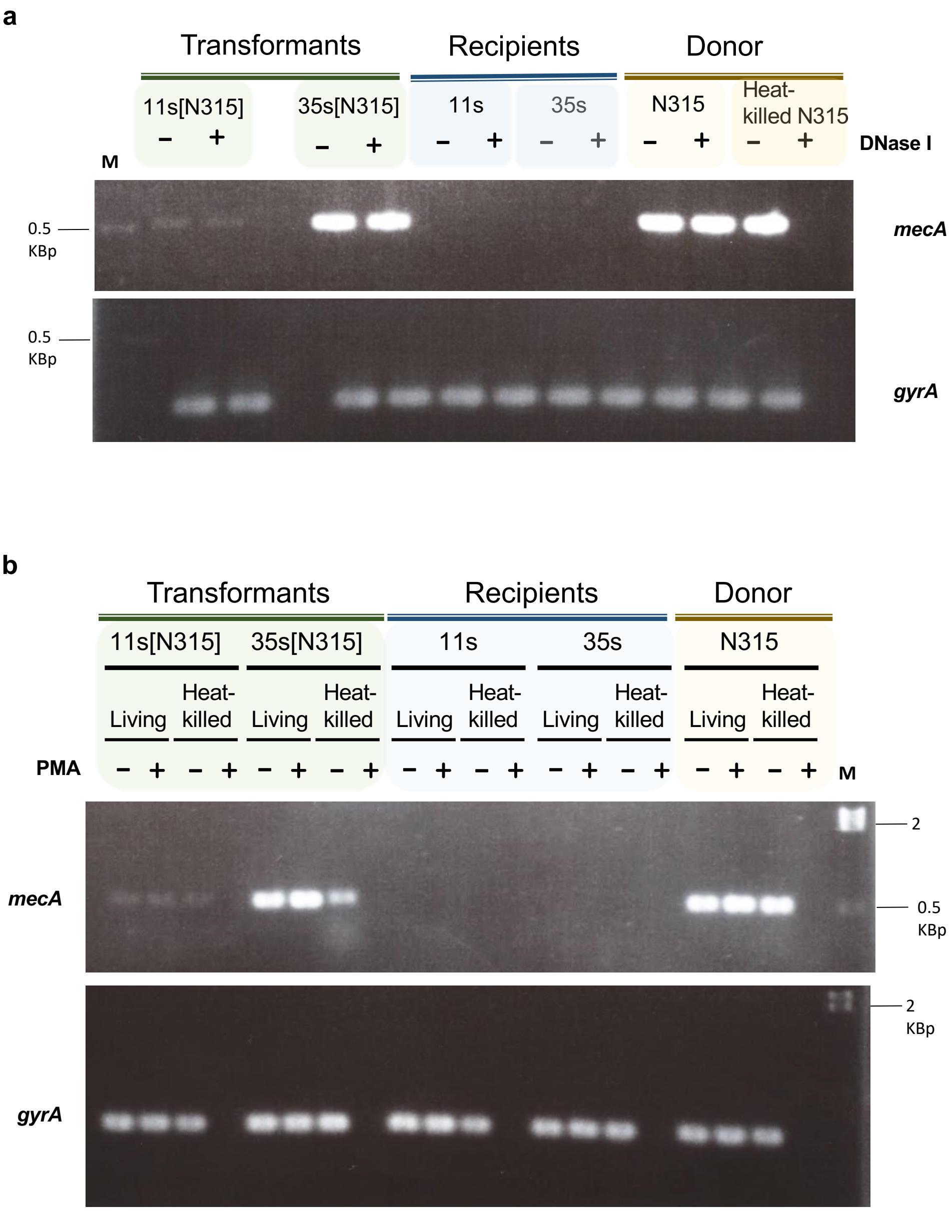

Supplementary Figure 15. **Confirmation of *mecA* incorporation into cells.**
Living and heat-killed cells were treated with 50 μg mL^{-1} DNase I for 3 hours at 37 ˚C (**a**) or with 50 μM PMA (Propidium monoazide, Biotium, CA) (**b**), followed by genome extraction. PCR for *mecA* and *gyrA* is shown. M: DNA size marker, λ Hind**III**.
DNase I and PMA treatment abolished PCR amplification in heat-killed cells but were unable to abolish *mecA* PCR signals from unstable transformants, such as 11s[N315], confirming that *mecA* is incorporated into cells. Each experiment was repeated at least twice independently with similar results.
Source data are provided as a Source Data file.

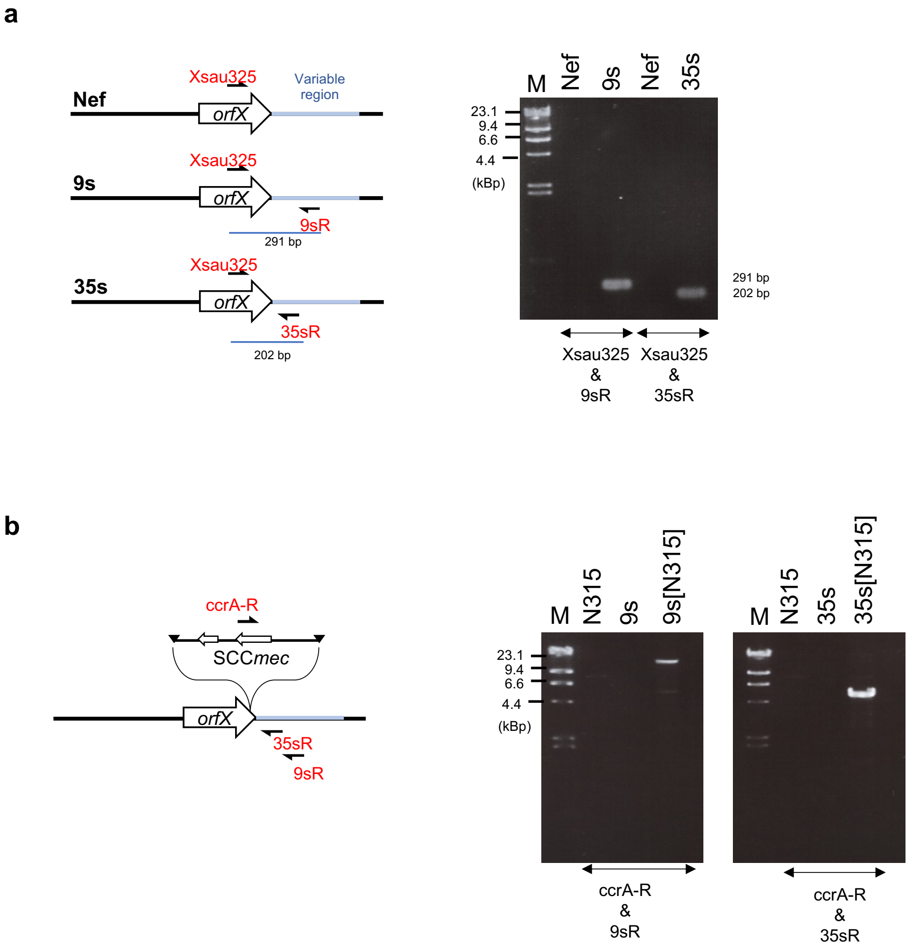

Supplementary Figure 16. Confirmation of site-specific integration of SCC*mec* in transformants.

a, Primers 9sR and 35sR are specific for strains 9s and 35s, respectively, and Nef does not have these sequences. The primer Xsau325 is compatible for Nef (as shown in Figure 4) as well as for 9s and 35s.

b, The primer ccrA-R is designed for the *ccrA* region in SCC*mec* type II in N315. M: DNA size marker, λ HindIII.

The experiment was repeated at least twice independently with similar results.

The PCR with 9sR or 35sR primer, together with ccrA-R, confirmed the site-specific integration of SCC*mec* in the chromosome of transformants.

Source data are provided as a Source Data file.

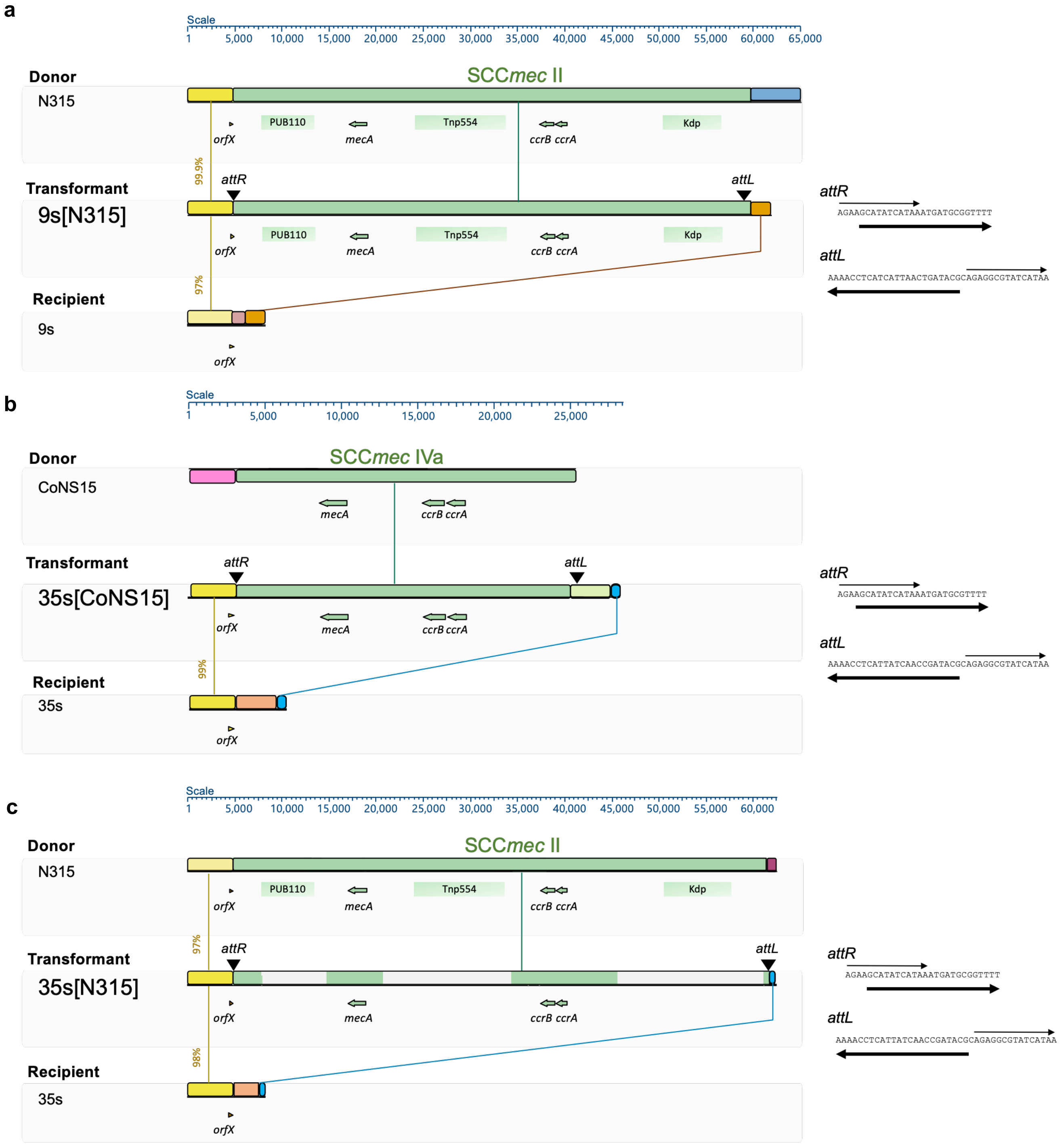

Supplementary Fig. 17. Confirmation of SCC integration into chromosome by genome sequencing. Sequenced genome contigs of transformants (**a,** 9s[N315], **b,** 35s[CoNS15], **c,** 35s[N315]) were aligned with known sequences or contigs of donors, and recipients. SCC*mec* region is shown by green. Homologous regions are shown by similar colored blocks and are connected by lines. Upstream regions of transformants 35s[CoNS15] and 35s[N315] were identical to the original recipient sequences, but the upstream region of 9s[N315] was more similar to the donor, implicating additional homologous recombination. The percent identity of the aligned upstream regions (yellow blocks) is shown. In all cases, downstream regions in transformants were identical to the original recipient sequences. The *att* sequences detected in transformants are shown on the right. Although insertion boundaries were sometimes rearranged (e.g. 35s[CoNS15]; pale green region), chromosomal backbones of transformants were identical to the recipients, which was also confirmed by multiplex PCR (Supplementary Fig. 18).

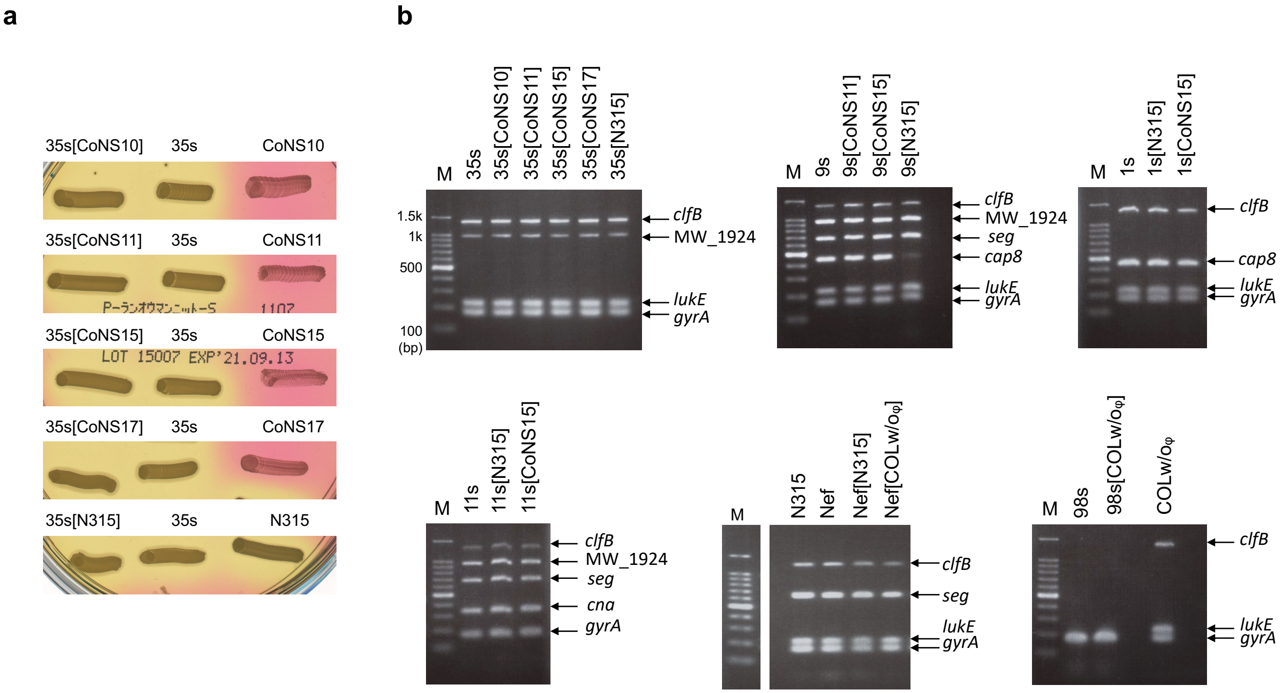

Supplementary Figure 18. Validation of the SCC*mec* transformants.
a, 35s transformants and CoNS donors were replicated onto Mannitol-Salt agar. Mannitol utilization is represented by the yellow color of the medium. Transformants were positive for mannitol utilization while CoNS donors were negative, confirming that these transformants are *S. aureus*.
b, Transformants share the same genetic background as the recipient as validated by multiplex PCR: The amplification patterns are same between transformants and each original recipient. M: 100 bp DNA ladder (Takara). **a, b,** The experiments were repeated at least twice independently with similar results.
Source data are provided as a Source Data file.

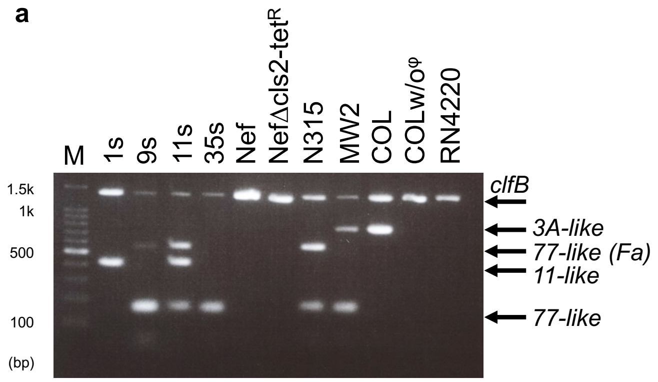

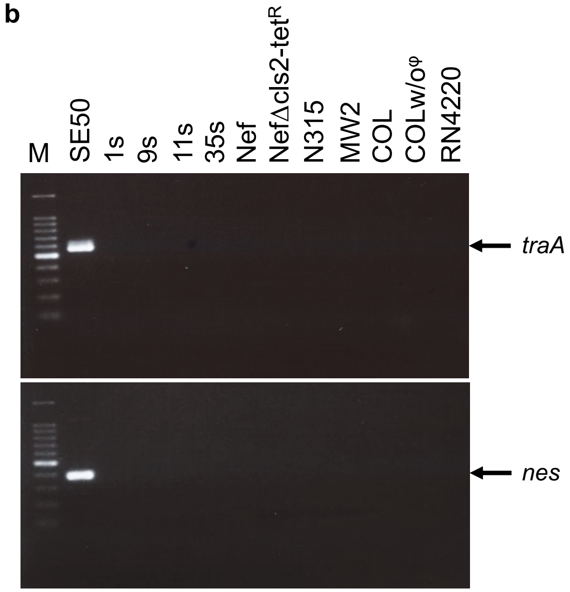

Strain	clfB	Phage PCR product size (bp)	Phage type *	Serogroup *	Coding sequence *
1s	+	405	11-like phages	B	hypothetical tail proteins
9s	+	548	77-like phages (Fa)	F	packaging proteins
		155	77-like phages	F	hypothetical tail proteins
11s	+	548	77-like phages (Fa)	F	packaging proteins
		405	11-like phages	B	hypothetical tail proteins
		155	77-like phages	F	hypothetical tail proteins
35s	+	155	77-like phages	F	hypothetical tail proteins
Nef	+	No product			
NefΔcls2-tet^R	+	No product			
N315	+	548	77-like phages (Fa)	F	packaging proteins
		155	77-like phages	F	hypothetical tail proteins
MW2	+	744	3A-like phages	A	tail fibers
		155	77-like phages	F	hypothetical tail proteins
COL	+	744	3A-like phages	A	tail fibers
COLw/oφ	+	No product			

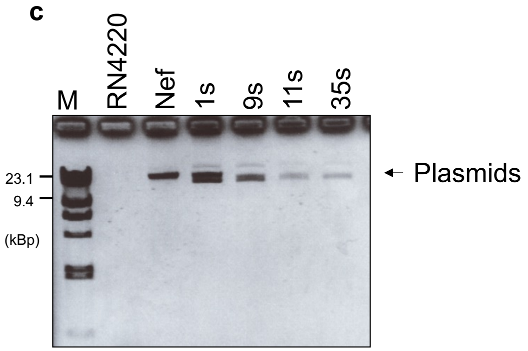

Supplementary Figure 19. Presence of phages, conjugative elements, and plasmids. Chromosomal DNA of donors and recipients were tested for presence of phage (**a**) or conjugative elements (**b**). RN4220 was used as negative control. SE50 was used as positive control for presence of conjugative elements (*traA* and *nes*). M: 100bp marker (Takara). **c,** Plasmid DNA was purified from log-phase cells. Transformable MSSA isolates (Nef, 1s, 9s, 11s, 35s) have plasmids. RN4220 has no plasmid and was used as a negative control. M: DNA size marker, λ HindIII.

To confirm the absence of conjugative elements (including *traA* and *nes*), PCR using the method and primers described by Cafini et al., 2016 was conducted[1].

*Phages were detected using the multiplex PCR method described by Pantůček et al.,2004[2].

Source data are provided as a Source Data file.

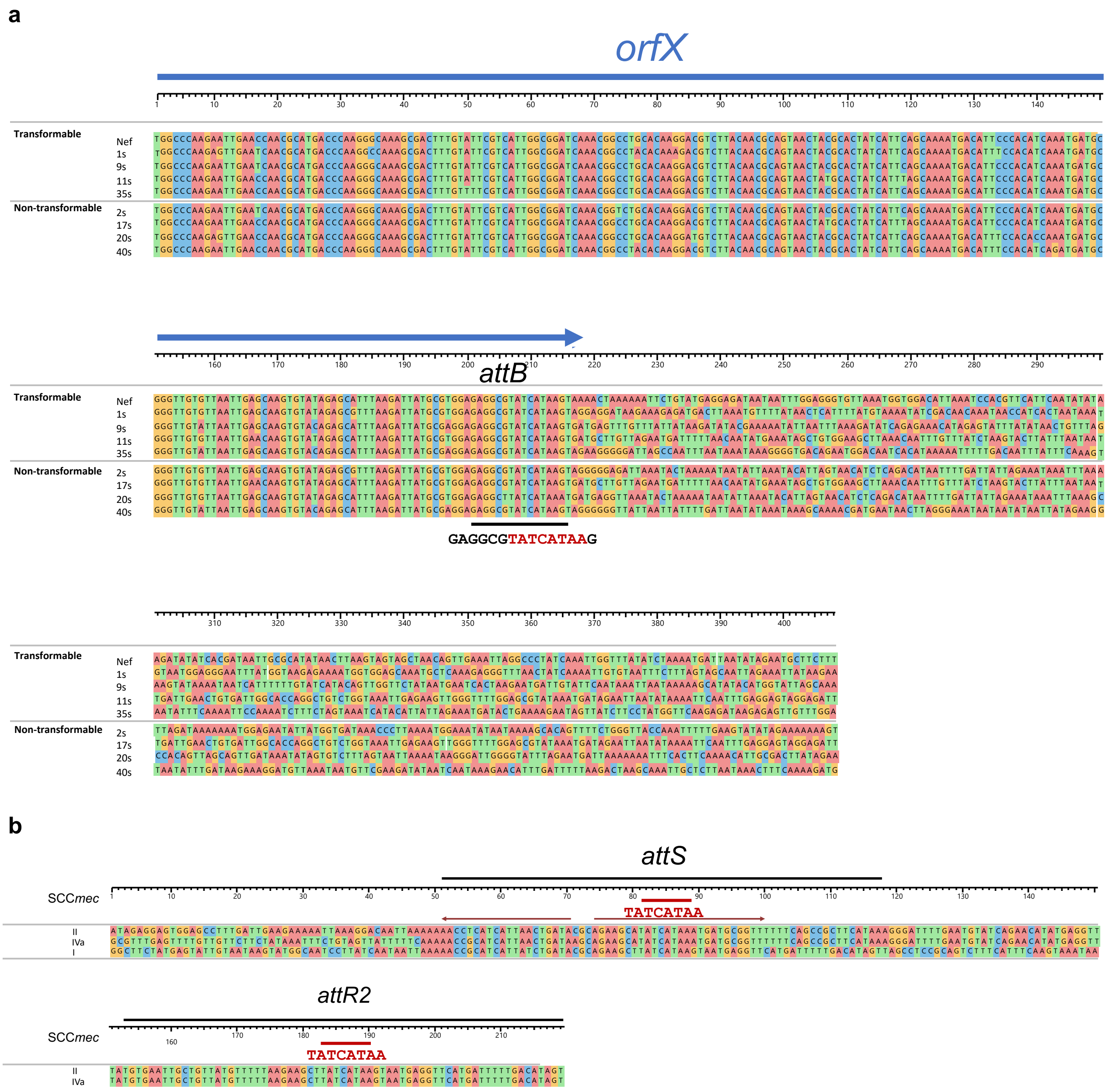

Supplementary Figure 20. ***att*** **sequences in recipients and donors.**

a, Sequences encompassing *attB* site in MSSA strains. The 15 bp core sequence of the *attB* attachment site is shown below the alignment.

b, The *attS* and *attR2* sequences of SCC*mec*[3]. Type II (N315), Type IVa (MW2), and Type I (COL) share conserved *attS* core sequence (TATCATAA), which is also found in the other *att* sites. The *attR2* in SCC*mec* II and IVa can be used for SCC excision but it can also affect the insertion frequency[3].

Supplementary Table 1. MIC (μg mL^{-1}) of cell wall-targeting antibiotics in CS2, TSB, or MH.

	Vancomycin			Bacitracin		Nisin	
	CS2	TSB	MH	CS2	TSB	CS2	TSB
Nef	1	2	1	64	> 256	64	128
Δ12	0.25	0.5	0.25	-	-	-	-
Δ12(pHY-12)	1	1	1	-	-	-	-
Δ12(pHY)	0.25	0.5	0.5	-	-	-	-
Δ17	-	-	-	4	8	8	16
Δ17(pHY-17)	-	-	-	32	256	32	64
Δ17(pHY)	-	-	-	4	8	8	16

Supplementary Table 2. MSSA clinical isolates screened for *mecA*-transformability.

Screened by using MR-CoNS8 as donor					Screened by using MR-CoNS3 as donor					Screened by using COLw/oφ as donor**								
isolate	CC	*blaZ*	*blaI*	Result*	isolate	CC	*blaZ*	*blaI*	Result*	isolate	CC	Result*	isolate	CC	Result*	isolate	CC	Result*
**1s**	133	+	+	**+**	**21s**	8	+	+	–	**41s**	1	–	**61s**	untypable	△/–/△	**81s**	1	△/–/–
**2s**	1	–	–	–	**22s**	5	+	+	–	**42s**	8	–	**62s**	1	△/–/–	**82s**	45	△/–/△
**3s**	1	–	–	–	**23s**	45	–	–	–	**43s**	133	–	**63s**	30	△/–/△	**83s**	8	–
**4s**	59	+	+	–	**24s**	5	+	+	–	**44s**	1	–	**64s**	1	△/–/–	**84s**	8	**+**
**5s**	133	–	–	–	**25s**	1	–	–	–	**45s**	45	–	**65s**	1	△/–/△	**85s**	133	–
**6s**	untypable	–	–	–	**26s**	untypable	–	–	–	**46s**	8	–	**66s**	1	–	**86s**	133	–
**7s**	1	–	–	–	**27s**	1	+	+	–	**47s**	30	–/△/–	**67s**	5	△/–/–	**87s**	1	–
**8s**	8	+	+	–	**28s**	133	+	+	–	**48s**	1	–	**68s**	30	△/–/–	**88s**	133	△/△/△
**9s**	untypable	+	+	**+**	**29s**	133	+	+	–	**49s**	45	–/△/–	**69s**	30	–	**89s**	5	–
**10s**	5	+	+	–	**30s**	5	+	+	–	**50s**	untypable	△/△/–	**70s**	untypable	△/–/△	**90s**	133	–
**11s**	45	+	+	**+**	**31s**	1/30	–	–	–	**51s**	untypable	–	**71s**	untypable	△/△/△	**91s**	8	–
**12s**	1	–	–	–	**32s**	5	+	+	△	**52s**	8	△/△/–	**72s**	8	–	**92s**	8	–
**13s**	untypable	–	–	–	**33s**	1/30	+	+	–	**53s**	133	–	**73s**	8	**+**	**93s**	8	–
**14s**	1	–	–	–	**34s**	133	–	–	–	**54s**	untypable	–	**74s**	133	△/△/△	**94s**	8	–
**15s**	8	–	–	–	**35s**	8	+	+	**+**	**55s**	133	–	**75s**	untypable	–	**95s**	30	–
**16s**	30	+	+	–	**36s**	8	–	–	–	**56s**	45	△/–/–	**76s**	133	△/△/–	**96s**	133	–
**17s**	45	–	–	–	**37s**	45	–	–	–	**57s**	30	△/△/–	**77s**	45	△/△/–	**97s**	8	–
**18s**	59	–	–	–	**38s**	8	+	+	–	**58s**	untypable	△/–/–	**78s**	45	△/△/△	**98s**	untypable	**+**
**19s**	30	+	+	–	**39s**	1	–	–	△	**59s**	5	–	**79s**	1	△/–/△	**99s**	133	–
**20s**	untypable	–	–	–	**40s**	1	+	+	–	**60s**	30	–	**80s**	8	△/–/–			

* +: Transformation confirmed by mecA PCR after replica (including unstable transformation)
-: No colony emerged in the cefmetazole selection, or emerged colony could not grow on replica.
△: Colonies emerged in the selection by cefmetazole and could grow on replica, but not further tested by PCR.

** Three independent transformation experiments were done. e.g. Δ/-/- : colony emerged in 1st screening, but none in 2nd and 3rd.
Low reproducibility is probably because of the low transformation efficiency close to the detection limit.

20 MSSA (1s~20s) were tested by using MR-CoNS8 as a donor. 20 MSSA (21s~40s) and 59 MSSA (41s~99s) were by MR-CoNS3 and COLw/o$_{\varphi}$, respectively. In each experiment, Nef$_{\Delta}$comE was used as a negative control. CC: clonal complex. The presence of *blaZ* and *blaI* that has a role in the regulation of *mecA* expression (see Discussion) was tested by PCR. Strains selected for detail analysis are marked (1s, 9s, 11s, 35s, and 98s).

Supplementary Table 3. Intra and interspecies transformation of distinct SCC*mec* elements in biofilm growth conditions. (<u>Number of transformants obtained</u>).

3-day biofilm in CS2

Selection: cefmetazole

	**Heat-killed donor**	**SCC type**	**Recipient** Nef	NefΔcomE	NefattB*	1s	9s	9sΔcomE	11s	35s	98s
S. aureus	COL	I	**7** (n = 1), ND (n = 1)	ND (n = 2)		**3** (n = 2)	**9.5** (n = 2)		**8** (n = 2)	**4** (n = 2)	
	COLw/oφ	I	**3** (n = 2)	ND (n = 4)		**3.5** (n = 2)	**3** (n = 2)		**2.5** (n = 2)	**4** (n = 2)	**4** ± 2.6 (n = 3)
	N315	II	**21** ± 16 (n = 5)	ND (n = 4)	ND (n = 6)	**11** ± 9.9 (n = 5)	**5.3** ± 5.3 (n = 6), ND (n = 2)	ND (n = 4)	**4.6** ± 3.6 (n = 5)	**10** ± 5.9 (n = 7)	
	N315ΔccrAB	II (ΔccrAB)	ND (n = 4)	ND (n = 6)	ND (n = 3)		ND (n = 3)	ND (n = 3)			
	Nef	none		ND (n = 2)	ND (n = 4)	ND (n = 3)	ND (n = 3)		ND (n = 3)	ND (n = 3)	
	35s[CoNS17]	IVa	**3** (n = 1), ND (n = 1)	ND (n = 2)		**14** (n = 2)	**5.5** (n = 2)		**1** (n = 1), ND (n = 1)	**3.5** (n = 2)	
	MW2	IVa	**4.5** (n = 2)	ND (n = 2)		**11** (n = 2)	**21** (n = 2)		**2** (n = 2)	**10** (n = 2)	
MR-CoNS	CoNS16	I	**4** (n = 1), ND (n = 1)	ND (n = 5)	ND (n = 3)	**8** (n = 2)	**19** (n = 1), ND (n = 1)		**5** (n = 2)	**19** ± 29.5 (n = 3)	
	CoNS9	III	**26** (n = 2)	ND (n = 2)		**23** (n = 1)	**20** ± 5.5 (n = 3)		**29** (n = 1)	**11** (n = 2)	
	CoNS10	IVa	**82** (n = 2)	ND (n = 5)	ND (n = 3)	**13** (n = 1)	**27** (n = 1)		**12** ± 9.1 (n = 3)	**10** (n = 2)	
	CoNS11	IVa	**47** ± 65.6 (n = 3)	ND (n = 5)	ND (n = 4)	**20** (n = 2)	**7** ± 1.2 (n = 3)	ND (n = 2)	**16** (n = 2)	**14** (n = 2)	
	CoNS15	IVa	**5** (n = 2)	ND (n = 5)	ND (n = 3)	**9** (n = 2)	**10** (n = 2)		**6** (n = 2)	**11** (n = 2)	
	CoNS17	IVa	**9** (n = 2)	ND (n = 5)	ND (n = 3)	**13** ± 7.9 (n = 5)	**13** ± 3.6 (n = 4)		**14** (n = 1)	**8** (n = 2)	

The mean of **number of transformants** obtained from single well is shown with ± SD.
n: number of independent experiments.
ND: none detected (0)

Supplementary Table 4. MIC (μg mL^{-1}) of cefmetazole and cefoxitin in TSB.

	strain	Cefmetazole	Cefoxitin
MSSA recipients	Nef	4	4
	1s	4	4
	9s	4	4
	11s	4	4
	35s	4	4
MRSA donors	N315	8	8
	MW2	16	16
MR-CoNS donors	CoNS10	16	16
	CoNS11	64	64
	CoNS15	64	64
	CoNS17	32	32
Transformants (Recipient[Donor])	35s[N315]	64	64
	35s[CoNS10]	64	64
	35s[CoNS11]	64	64
	35s[CoNS15]	64	64
	35s[CoNS17]	64	64
	1s[CoNS11]	8	8
	1s[CoNS15]	8	8
	9s[CoNS11]	8	8
	9s[CoNS15]	8	8
	11s[CoNS11]	8	8
	11s[CoNS15]	8	8

Supplementary Table 5. Bacterial strains and plasmids used in this study.

Strain or plasmid	Description	Source
**Strains**		
RN4220	Derivative of 8325-4, restriction minus, modification plus	4
N315	Pre-MRSA, SCC*mec* II, Km^R, Erm^R, clonal complex 5, carrying *blaI* and *blaZ* in plasmid	5
N315ex	N315 cured of SCC*mec*, Km^S, Erm^R	6
N315ΔccrAB	N315 lacking *ccrAB* locus	This study
N315Δcls2-tet^R	N315Δcls2 mutant, Tet^R	7
Nef	N315ex w/oφ (N315ex cured of the φN315 prophage)	8
Nef-GFP	Nef carrying pMK3-com-gfp	This study
Nef-NH7-GFP	Nef carrying pRIT-sigHNH7 and pMK3-com-gfp	This study
Nef-pRIT5H	Nef carrying pRIT5H	8
Nef-pRITcomGFP	Nef carrying pRIT-com-gfp	This study
Nef-pT181	Nef carrying pT181	This study
NefattB*	Nef with mutated *attB* site	This study
Nef-H	Nef carrying pRIT-sigH	8
Nef-H-GFP	Nef-H carrying pMK3-com-gfp	This study
Nef-V-GFP	Nef carrying pRIT5H and pMK3-com-gfp	This study
NefΔcls2-tet^R	NefΔcls2 mutant, Tet^R (transduction of Tet^R from N315Δcls2)	This study
NefΔcomE	Nef lacking *comE* operon	8
NefΔcomG	Nef lacking *comG* operon	8
ΔH	Nef lacking *sigH*	This study
ΔH-GFP	ΔH carrying pMK3-com-gfp	This study
COLw/oφ	COL strain cured of the φL54a prophage, clonal complex 8	8
1s	MSSA clinical isolate, clonal complex 133	This study
9s	MSSA clinical isolate, clonal complex untypable	This study
9sΔcomE	9s mutant lacking *comE* operon	This study
11s	MSSA clinical isolate, clonal complex 45	This study
35s	MSSA clinical isolate, clonal complex 8	This study
98s	MSSA clinical isolate, clonal complex untypable	This study
MR-CoNS3	Methicillin resistant *S. epidermidis*, SCC*mec* nontypeable	This study
MR-CoNS8	Methicillin resistant *S. lugdunensis*, SCC*mec* nontypeable	This study
MR-CoNS9	Methicillin resistant *S. epidermidis*, SCC*mec* III	This study
MR-CoNS10	Methicillin resistant *S. epidermidis*, SCC*mec* IVa	This study
MR-CoNS11	Methicillin resistant *S. epidermidis*, SCC*mec* IVa / I	This study
MR-CoNS15	Methicillin resistant *S. epidermidis*, SCC*mec* IVa	This study
MR-CoNS16	Methicillin resistant *S. caprae*, SCC*mec* I	This study
MR-CoNS17	Methicillin resistant *S. epidermidis*, SCC*mec* IVa	This study
r3	MRSA clinical isolate, SCC*mec*II, clonal complex 5	9
r3-H	r3 carrying pRIT-sigH	This study
r3-pRITcomGFP	r3 carrying pRIT-com-gfp	This study
r408	MRSA clinical isolate, SCC*mec*II, clonal complex 5	9
r408-H	r408 carrying pRIT-sigH	This study
r408-pRITcomGFP	r408 carrying pRITcomGFP	This study
r59	MRSA clinical isolate, SCC*mec*II, clonal complex 5	9
r59-H	r59 carrying pRIT-sigH	This study
r59-pRITcomGFP	r59 carrying pRIT-com-gfp	This study
s142	MSSA clinical isolate	9
s142-H	s142 carrying pRIT-sigH	This study
s142-pRITcomGFP	s142 carrying pRIT-com-gfp	This study
s1567	MSSA clinical isolate, clonal complex 5	9
s1567-H	s1567 carrying pRIT-sigH	This study
s1567-pRITcomGFP	s1567 carrying pRIT-com-gfp	This study
Δ3	Nef lacking *TCS3*	This study
Δ3-GFP	Δ3 carrying pMK3-com-gfp	This study
Δ4	Nef lacking *TCS4*	This study
Δ4-GFP	Δ4 carrying pMK3-com-gfp	This study
Δ5	Nef lacking *TCS5*	This study
Δ5-GFP	Δ5 carrying pMK3-com-gfp	This study
Δ6	Nef lacking *TCS6*	This study
Δ6-GFP	Δ6 carrying pMK3-com-gfp	This study
Δ7	Nef lacking *TCS7*	This study
Δ7-GFP	Δ7 carrying pMK3-com-gfp	This study

Supplementary Table 5. Bacterial strains and plasmids used in this study (continued).

Strain or plasmid	Description	Source
**Strains**		
Δ8	Nef lacking *TCS8*	This study
Δ8-GFP	Δ8 carrying pMK3-com-gfp	This study
Δ9	Nef lacking *TCS9*	This study
Δ9-GFP	Δ9 carrying pMK3-com-gfp	This study
Δ10	Nef lacking *TCS10*	This study
Δ10-GFP	Δ10 carrying pMK3-com-gfp	This study
Δ11	Nef lacking *TCS11*	This study
Δ11-GFP	Δ11 carrying pMK3-com-gfp	This study
Δ12	Nef lacking *TCS12*	This study
Δ12-GFP	Δ12 carrying pMK3-com-gfp	This study
Δ12(pHY-12)-GFP	Δ12 carrying pMK3-com-gfp and pYH-12 (TCS12 complementary strain)	This study
Δ12(pHY)-GFP	Δ12 carrying pMK3-com-gfp and pYH300PLK (empty vector)	This study
Δ13	Nef lacking *TCS13*	This study
Δ13-GFP	Δ13 carrying pMK3-com-gfp	This study
Δ13(pHY-13)-GFP	Δ13 carrying pMK3-com-gfp and pYH-13 (TCS13 complementary strain)	This study
Δ13(pHY)-GFP	Δ13 carrying pMK3-com-gfp and pYH300PLK (empty vector)	This study
Δ13(TCS13)	Δ13 with chromosomal *TCS13* complementation	This study
Δ13(TCS13)-GFP	Δ13 with chromosomal *TCS13* complementation carrying pMK3-com-gfp	This study
Δ13-NH7	Δ13 carrying pRIT-sigHNH7	This study
Δ13-NH7-GFP	Δ13 carrying pRIT-sigHNH7 and pMK3-com-gfp	This study
Δ13H	Δ13 carrying pRIT-sigH	This study
Δ13-V	Δ13 carrying pRIT5H	This study
Δ13-V-GFP	Δ13 carrying pRIT5H and pMK3-com-gfp	This study
Δ14	Nef lacking *TCS14*	This study
Δ14-GFP	Δ14 carrying pMK3-com-gfp	This study
Δ15	Nef lacking *TCS15*	This study
Δ15-GFP	Δ15 carrying pMK3-com-gfp	This study
Δ16	Nef lacking *TCS16*	This study
Δ16-GFP	Δ16 carrying pMK3-com-gfp	This study
Δ17	Nef lacking *TCS17*	This study
Δ17(TCS17)	Δ17 with chromosomal *TCS17* complementation	This study
Δ17(TCS17)-GFP	Δ17 with chromosomal *TCS17* complementation carrying pMK3-com-gfp	This study
Δ17-GFP	Δ17 carrying pMK3-com-gfp	This study
Δ17(pHY-17)-GFP	Δ17 carrying pMK3-com-gfp and pYH-17 (TCS17 complementary strain)	This study
Δ17(pHY)-GFP	Δ17 carrying pMK3-com-gfp and pYH300PLK (empty vector)	This study
Δ17-NH7	Δ17 carrying pRIT-sigHNH7	This study
Δ17-NH7-GFP	Δ17 carrying pRIT-sigHNH7 and pMK3-com-gfp	This study
Δ17-H	Δ17 carrying pRIT-sigH	This study
Δ17-V	Δ17 carrying pRIT5H	This study
Δ17-V-GFP	Δ17 carrying pRIT5H and pMK3-com-gfp	This study
Nef-pHY-P_{comG}-*gfp*-P_{comE}-*dsRed*	Nef carrying pHY-PcomGgfp-PcomEdsRed	This study
ΔH-pHY-P_{comG}-*gfp*-P_{comE}-*dsRed*	ΔH carrying pHY-PcomGgfp-PcomEdsRed	This study
SE50	*S. epidermidis*, carries *cfr* pSCFS7-like plasmid and conjugative genes	1
DH5α	*E. coli* plasmid cloning host	Toyobo Ltd., Japan
**Plasmids**		
pMADtet	pMAD derivative, Amp^R (*E. coli*), Erm^R, Tet^R (*S. aureus*)	8
pMADtet-Δ3	Vector for deletion of *TCS3* locus, Amp^R (*E. coli*), Erm^R, Tet^R (*S. aureus*)	This study
pMADtet-Δ4	Vector for deletion of *TCS4* locus, Amp^R (*E. coli*), Erm^R, Tet^R (*S. aureus*)	This study
pMADtet-Δ5	Vector for deletion of *TCS5* locus, Amp^R (*E. coli*), Erm^R, Tet^R (*S. aureus*)	This study
pMADtet-Δ6	Vector for deletion of *TCS6* locus, Amp^R (*E. coli*), Erm^R, Tet^R (*S. aureus*)	This study
pMADtet-Δ7	Vector for deletion of *TCS7* locus, Amp^R (*E. coli*), Erm^R, Tet^R (*S. aureus*)	This study
pMADtet-Δ8	Vector for deletion of *TCS8* locus, Amp^R (*E. coli*), Erm^R, Tet^R (*S. aureus*)	This study
pMADtet-Δ9	Vector for deletion of *TCS9* locus, Amp^R (*E. coli*), Erm^R, Tet^R (*S. aureus*)	This study
pMADtet-Δ10	Vector for deletion of *TCS10* locus, Amp^R (*E. coli*), Erm^R, Tet^R (*S. aureus*)	This study
pMADtet-Δ11	Vector for deletion of *TCS11* locus, Amp^R (*E. coli*), Erm^R, Tet^R (*S. aureus*)	This study
pMADtet-Δ12	Vector for deletion of *TCS12* locus, Amp^R (*E. coli*), Erm^R, Tet^R (*S. aureus*)	This study
pMADtet-Δ13	Vector for deletion of *TCS13* locus, Amp^R (*E. coli*), Erm^R, Tet^R (*S. aureus*)	This study
pMADtet-Δ14	Vector for deletion of *TCS14* locus, Amp^R (*E. coli*), Erm^R, Tet^R (*S. aureus*)	This study
pMADtet-Δ15	Vector for deletion of *TCS15* locus, Amp^R (*E. coli*), Erm^R, Tet^R (*S. aureus*)	This study
pMADtet-Δ16	Vector for deletion of *TCS16* locus, Amp^R (*E. coli*), Erm^R, Tet^R (*S. aureus*)	This study
pMADtet-Δ17	Vector for deletion of *TCS17* locus, Amp^R (*E. coli*), Erm^R, Tet^R (*S. aureus*)	This study
pMADtet-ΔH	Vector for deletion of *sigH* locus, Amp^R (*E. coli*), Erm^R, Tet^R (*S. aureus*)	This study
pMADtet-ΔccrAB	Vector for deletion of *ccrAB* locus, Amp^R (*E. coli*), Erm^R, Tet^R (*S. aureus*)	This study
pMADtetcomEII	Vector for deletion of *comE* locus, Amp^R (*E. coli*), Erm^R, Tet^R (*S. aureus*)	8
pMADtet-TCS13	Vector for complementation of *TCS13* locus, Amp^R (*E. coli*), Erm^R, Tet^R (*S. aureus*)	This study
pMADtet-TCS17	Vector for complementation of *TCS17* locus, Amp^R (E. coli), Erm^R, Tet^R (S. aureus)	This study
pMADtet-attB*	Vector for mutational substitution of *attB* locus, Amp^R (*E. coli*), Erm^R, Tet^R (*S. aureus*)	This study
pHY-P_{comG}-*gfp*-P_{comE}-*dsRed*	P_{comG}-*gfp* transcriptional fusion and P_{comE}-*dsRed* transcriptional fusion in pHY300PLK, Tet^R (*E. coli*, *S. aureus*)	This study
pMK3-com-gfp	P_{comG}-*gfp* transcriptional fusion in pMK3, Amp^R (*E. coli*), Tet^R *(S. aureus)*	8
pRIT5H	Shuttle vector, ori-pC194, AmpR (*E. coli*), CmR (*S. aureus*), P*spa*-MCS	9
pRIT-sigHNH7	pRIT5H carrying *sigH* gene in MCS, *sigH* mRNA overexpressing plasmid	8
pRIT-sigH	pRIT5H carrying *sigH* gene with SigA-type translation initiation sequences, allowing constitutive expression of SigH	8
pRIT-com-gfp	P*comG-gfp* transcriptional fusion in pRIT	8
pHY300PLK	Shuttle vector, ori-pAMα1, Amp^R (*E.coli*), Tet^R (*S. aureus*)	Takara, Japan
pHY-12	pHY300PLK-TCS12, TCS12 complementation plasimd	This study
pHY-13	pHY300PLK-TCS13, TCS12 complementation plasmid	This study
pHY-17	pHY300PLK-TCS17, TCS17 complementation plasmid	This study

Supplementary Table 6. List of primers used in this study.

Primer	Sequence	Source
**ΔTCS construction (pMADtet targetting vector construction)**		
TCS3-A	GGAGGATCCAGAAGATTTTGTTCCCTATC	This study
TCS3-B	CATTGAATCATCTCCAAAAATTTATGATG	This study
TCS3-C	TTTTTGGAGATGATTCAATGAAATAGATTAGCACATAACTAATGATTATG	This study
TCS3-D	GGAGTCGACGTGTATCATTGACTGGTTTTG	This study
TCS4-A	GGAGGATCCGGCATGTTAGAGCATATGC	This study
TCS4-B	CACGATAGCACCTCAGTGAATT	This study
TCS4-C	GAGGTGCTATCGTGCTTTAACAGTAATCCTTTTTTTTATGCATTTTAC	This study
TCS4-D	GGAGTCGACACAAATAGCGGTGCGAATAAG	This study
TCS5-A	GGAGGATCCGAAACAATTGAACGCCTAGG	This study
TCS5-B	CATCCATATCACCCAATATCATTTAG	This study
TCS5-C	GATATTGGGTGATATGGATGTAAACATGCGTTTTGTTACTTAGAATTG	This study
TCS5-D	GGAGTCGACTTTGTCCACCAGACAATTCAG	This study
TCS6-A	GGAGGATCCAGCTCAAACACTTCCTGTTC	This study
TCS6-B	CATCTATTTTTTCACCTCTGTTCTTACG	This study
TCS6-C	GAGGTGAAAAAATAGATGTCATAATCCGATTTATTTATAAAATAAAATGC	This study
TCS6-D	GGAGTCGACTTTTAAGCCAAAGAGCTG	This study
TCS7-A	GGAGGATCCATTGCCGGAAGATGTTAAACC	This study
TCS7-B	CATATTTTATTCCGCCCTTTTAAAATGAC	This study
TCS7-C	GGGCGGAATAAAATATGATCTAAATACAAACAAAAAAGTATTGAGTG	This study
TCS7-D	GGAGTCGACGCAATAAATGTATCACCATCC	This study
TCS8-A	GGAGGATCCCGCATGATTACGCTATTTTAG	This study
TCS8-B	CATTTGTACACCTCATATTACGACTTTTTC	This study
TCS8-C	GTAATATGAGGTGTACAAATGTTTTAATCATGTCTGAGACGTCAATC	This study
TCS8-D	GGAGTCGACACCTTCCAAAGTCTGTTGATC	This study
TCS9-A	GGAGGATCCGATGATAGAGGACGTACAG	This study
TCS9-B	CAGGTCATACCTCCCACAC	This study
TCS9-C	GAGGTATGACCTGTATGGAATAAAACTGAATATAGTTATTTAAGAACGC	This study
TCS9-D	GGAGTCGACGTCATTACTGCGCATTGATC	This study
TCS10-A	GGAGGATCCGCAACACATGGTACAGCTC	This study
TCS10-B	CATGGTATGCCTCCCTAACTTATAA	This study
TCS10-C	GGAGGCATACCATGGAATAAAAATTAAAGTGGTAACAGCGC	This study
TCS10-D	GGAGTCGACCATTGTAAATCATCGTGCATG	This study
TCS11-A	GGAGGATCCGTCTTCTAACATGCGATCAAG	This study
TCS11-B	CATCAAATCGCTCCAATTCATTTTTAC	This study
TCS11-C	AATTGGAGCGATTTGATGATTTAGAATGAGCTTTTAAATATTTGTCG	This study
TCS11-D	GGAGTCGACTGTACCCATTACGAGTCTC	This study
TCS12-A	GGAGGATCCTCGTTCTATTATTGGGATGTG	This study
TCS12-B	CATCGATAAATCACCTCTACG	This study
TCS12-C	GAGGTGATTTATCGATGCAATAGTTCGTATCGAATTAAGAAAAG	This study
TCS12-D	GGAGTCGACACTTGGATTTGACGAACAAG	This study
TCS13-A	GGAGGATCCGCCACGTATCTTCAAAAGAG	This study
TCS13-B	CAATACGGCTCTACTTCCATAG	This study
TCS13-C	GAAGTAGAGCCGTATTGATATAATAAGATAATAAAGTCAGTTAACGGC	This study
TCS13-D	GGAGTCGACATGGTGCTGCCGTATATTTG	This study
TCS14-A	GGAGGATCCTGATTCACCGTTATAGTGTTG	This study
TCS14-B	CATAACCTTCACCTCGATAGC	This study
TCS14-C	CGAGGTGAAGGTTATGAAATAATTAAATAAAAAAGATCGCTGCC	This study
TCS14-D	GGAGTCGACGTCTGTGAGTAAAGGTGTATG	This study
TCS15-A	GGAGGATCCTATGCGCATACATTGTGTCG	This study
TCS15-B	CATAGCTATAAACTCCCTTATCTTTTTC	This study
TCS15-C	GGGAGTTTATAGCTATGCATTAATCTCTACCTCCTGAAAAAAC	This study
TCS15-D	GGAGTCGACTTTCATATTGATAAGCGCTCC	This study
TCS16-A	GGAGGATCCATTGATGAGTGGTGTGCC	This study
TCS16-B	CATGACTTACACCCTAATTCATC	This study
TCS16-C	TAGGGTGTAAGTCATGTTTTAGAGTTTGAAATTAATATAATTCAGTATAA	This study
TCS16-D	GGAGTCGACATGGTCATACGAAAGCATATC	This study
TCS17-A	GGAGGATCCAAATGATGGACCCATGCC	This study
TCS17-B	CATCTATAATCTTCTTCCTTCAATTG	This study
TCS17-C	GGAAGAAGATTATAGATGGAATAAAAACTTTCAATATTGTAAGTATACTA	This study
TCS17-D	GGAGTCGACAAGTGCACCTGTAGGTTC	This study

Supplementary Table 6. List of primers used in this study (continued).

Primer	Sequence	Source
**Confirmation of target deletion**		
TCS3-E	GTGTGTTATCAGAAACAATTGATC	This study
TCS3-F	GGTGCGTTAATCTTTGGTCC	This study
TCS4-E	GTCGAGTTAAAAGAAACATATGATAC	This study
TCS4-F	GGCCACACCAAATACCATAC	This study
TCS5-E	CATACCTGGGAGTCGTTATG	This study
TCS5-F	CTCTTGCAGCAGATGTTCG	This study
TCS6-E	GTTTGTTTAGCTTAAGCAACCC	This study
TCS6-F	CAATTTGATGATGGTGTTGGTG	This study
TCS7-E	TTATGATACTAAGTTACTTGAAAATCG	This study
TCS7-F	TATCAACCCCTATAAGCCTAAC	This study
TCS8-E	GTGAGAATCATTGTCAATTAGAAAC	This study
TCS8-F	TGATCTGAAACAATTCCTGCTG	This study
TCS9-E	CCAACTCAAGTGATAACAAGTG	This study
TCS9-F	CCATCATACTCATATCATCACC	This study
TCS10-E	CATTAATTATGAAACAGGTCATGC	This study
TCS10-F	CCATTAAGTGATGCAAATCCTAC	This study
TCS11-E	TGCCTTAACATTTGCTTTGTATATC	This study
TCS11-F	CGAAAATTGGTTGGTTATCTGG	This study
TCS12-E	ATGACACACAAATATATATCAACGC	This study
TCS12-F	CTGTAATTAGTCATTTCCTATTCG	This study
TCS13-E	TGAGGAGAGTGGTGTAAAATTG	This study
TCS13-F	TGTAGTCATTTATACGAAGGGAG	This study
TCS14-E	ACCTGATGCACTAGATGTAAAC	This study
TCS14-F	GATCTAAGGTTATGTAATTGGCC	This study
TCS15-E	CGTTATCGTTCAATAGCACAATG	This study
TCS15-F	AGTGAAAGGGACAAACCAATG	This study
TCS16-E	ATTTATGTTAAAACCAGATGCGTC	This study
TCS16-F	ATTTTAAACAAGACACTACAGTCAC	This study
TCS17-E	GGTCATTTCTTTGGCATGCG	This study
TCS17-F	TAACATCATCAATGCCTTTGACG	This study
**in *trans* complementation (pHY-12, 13, 17 construction)**		
TCS12CF	ATTCCCGGGAAAGAACAACTTAGCAAGTT	This study
TCS12CR	GTAGGATCCTTTTCTTAATTCGATACGAA	This study
TCS13CPrF	TACAAGCTTCAGTTAAGTATTTATTTCCT	This study
TCS13CR	AAAGGATCCCAAGATTTACAATTGAATAC	This study
TCS17CF	GTGGAATTCAAATATGAATCAAAAGCAGT	This study
TCS17CR	TCAGGATCCTGTTAGTTCTATATTAAAAC	This study
**Chromosomal complementation (pMADtet-TCS13, TCS17 construction)**		
TCS13-G	TATGGATCCAAGTGTATTTTTGTTTACCT	This study
TCS13-H	TAGGTCGACTAAGGTAAAACCTGTTGATA	This study
TCS17-G	ATGGGATCCTTGTCCCTTTTAAATATGAA	This study
TCS17-H	AATGTCGACGACGTTTTTGAATCAAGTG	This study
**ΔH mutant construction**		
H-A	GGAGGATCCGAATGCAATTAATAGATGCAATTGC	This study
H-B	CAAGTATTAAACTAACCCCTTCTATC	This study
H-C	GATAGAAGGGGTTAGTTTAATACTTGATTTGAAAAGCGCC	This study
H-D	GGAGTCGACTGTCTTCACTCCTCTAACC	This study
**ΔH mutant check**		
H-E	CGCCATTGCAAAAGAGAATTTAGAAG	This study
H-F	TATGCACTGCATACCAACGCTTTG	This study
**Construction of dual reporter plasmid**		
clpBterF	CCGGAATTCTAAAATTGAACCAAGAATG	This study
clpBterR	GTCATATCATGATAAACACATTAAATCATTTAAAAAGT	This study
PcomEF	AATGTGTTTATCATGATATGAC	This study
PcomER	GGATCCAAATCTTTATAGCGTAAT	This study
dsRedF	CGCTATAAAGATTTGGATCCGAGGAGGTTTATT	This study
dsRedR	ATTGTCGACCTGCAGTTACAGGAACAG	This study
pHYF	GAAGATCTCTGTCAGACCAAGTTTACTC	This study
pHYR	CGGGGTACCGACATTTCCCCGAAAAGTGC	This study
PcomGF	CGGGGTACCTATGTGTTCGATGAATTC	This study
gfpR	GAAGATCTCTATTTGTATAGTTCATCCATGCCATGTG	This study

Supplementary Table 6. List of primers used in this study (continued).

Primer	Sequence	Source
**SCC*mec* amplification**		
mecAF	GTAGTTGTCGGGTTTGGT	8
mecAR	GGTATCATCTTGTACCCA	8
3.0-R	CTCAGACAGCAATTTCCCG	8
ccrA-F	ACGTCAAAGTACGATGAAACAAC	8
ccrA-R	CTGACTTGTTCTCCAATGTTATCTG	8
Xsau325	GGATCAAACGGCCTGCACA	8
attL-F	ACTTATGATACGCCTCTGCTT	This study
attR-R	AGAAGCTTATCATAAGTAATGAGG	This study
ccrA.F	TGAATGCTTCACGCTTTGTC	This study
ccrA.R	TTGCGTTTGTTCTCTGAACG	This study
plsR	TACTCAAGGGAATGGCCAAG	This study
plsF	TCACACCATCTGCACCATTT	This study
ccrA1R	GATTGCGATGAAGTCGGTTT	This study
ccrA1F	ATCGAGGCATTAGCCAAAAA	This study
9sR	CACTAAGTAATATCTTGGTTGACATAC	This study
35sR	CAGGTAAGACAGTGGGGAAA	This study
**attB* mutant construction**		
attB-A	CGCGGATCCCCCAGCAGCGATGTTTGTAT	This study
attB-B	TTTAGTTTTACTT**G**TG**G**TA**A**GC**T**TCTCCACGCATAATCTTA	This study
attB-C	GCGTGGAGA**A**GC**T**TA**C**CA**C**AAGTAAAACTAAAAAATTCTGT	This study
attB-D	AATGTCGACCATTAAGCAGTTCATAATTGTCATCA	This study
**attB* mutant checck**		
attB-E	AAAATATTGGTATAATAAGAGG	This study
attB-F	AATGTCGACGCAAAATCAATTCCGAAGT	This study
**Amplification of *attB* flanking regions**		
orfXfor	GAGAAATATTGGAAGCAAGCC	10
unirev	GCACAGTGGGAATTAATCGAAGC	10
**ΔccrAB mutant construction**		
ccrAB-A	ATTCGCGGATCCGCAACACAGGCAATCGTATG	This study
ccrAB-B	GATAGCCTGTTTCTGTGCTGCAAGAGA	This study
ccrAB-C	CAGCACAGAAACAGGCTATCTAAGGGATTTGCAGATTCGAAGA	This study
ccrAB-D	TTACGCGTCGACATGCAGGTTGTTCTTGTTCATG	This study
**ΔccrAB mutant check**		
ccrAB-E	AAAAGTTGGCACAAGGCATC	This study
ccrAB-F	GTTGGAGCTCAGGTCGATTC	This study
***blaZ*, *blaI* check**		
blaZF	CAAGATGATATAGTTGCTTATTCTCC	11
blaZR	TGCTTGACCACTTTTATCAGC	11
blaIF	ATGACCAATAAGCAAGTTGAAA	12
blaIR	CATATCCCCTCCATACAGT	12
**CC typing by multiplex PCR**		
clfBF	AACAGAGCCAGCTTCAACAAATGA	13
clfBR	GTCTTTCGGATTTACTGCTGAATC	13
MW_1924F	TCACGAAGTCGAACGAAGAA	13
MW_1924R	GCTGAACGCTCTTCTGCTTC	13
segF	AGAATCAACAACTTTATTATCTCCG	13
segR	TATGTGAATGCTCAACCCGA	13
arcDF	TGCCATTGATGGATTAGCAA	13
arcDR	GTTTTTCAAGTGCTTGGGGA	13
cap8F	GGAGGAAATGACGATGAGGA	13
cap8R	TGTCACCCTGCTAGCATCAA	13
cnaF	TGCTGTCCACCTTGAATCTG	13
cnaR	GTTATTACGCCAGACGGAGC	13
lukEF	GCATTATGCTTTCTTTCTGCG	13
lukER	AATGGTCCAACAGGTTCAGC	13
gyrAF	AAGGTGTTCGCTTAATTCGC	14
gyrAR	ATTGCATTTCCTGGTGTTTC	14
**Phage typing by multiplex PCR**		
SGA1	TATCAGGCGAGAATTAAGGG	2
SGA2	CTTTGACATGACATCCGCTTGAC	2
SGB1	ACTTATCCAGGTGGYGTTATTG	2
SGB2	TGTATTTAATTTCGCCGTTAGTG	2
SGF1	CGATGGACGGCTACACAGA	2
SGF2	TTGTTCAGAAACTTCCCAACCTG	2
SGFa1	TACGGGAAAATATTCGGAAG	2
SGFa2	ATAATCCGCACCTCATTCCT	2
SGFb1	AGACACATTAAGTCGCACGATAG	2
SGFb2	TCTTCTCTGGCACGGTCTCTT	2
SGL1	GCTTAAAACAGTAACGGTGACAGTG	2
SGL2	TGCTACATCATCAAGAACACCTGG	2
SGD1	TGGGCTTCATTCTACGGTGA	2
SGD2	GTAATTTAATGAATCCACGAGAT	2
**Conjugation genes check**		
traAF	AATGGCCGAAGATAGAGAGGA	1
traAR	TTGCTATTTGTCCCGAGGCG	1
nesF	ATCATTGAATCTAGCCCAGCA	1
nesR	GGCACAGAAAACACGCTTGA	1
**Transformants check**		
pRIT5H1	GGAAGATCTGCGGCCGCGTCGACGGATCCCCGGGAAT	This study
pRIT5H2	TGCCACCTGACGTCTAAGAA	This study
TetR(EcoRI)	GGAATTCCTGTTATAAAAAAAGGATCAAT	8
TetF(salI)	CATATTGTCGACTAAGTGATGAAATACTG	8

Underlined, restriction site included in the oligonucleotide.
Bold, nucleotidic changes to cause silent mutations in *orfX*.

Supplementary Table 7. Composition of CS2 medium

	stock conc.	final conc.	1000 ml
**Sterile MilliQ water**			710ml
**Solution A**			100ml
Na_2HPO_4	71.4 g/L		
KH_2PO_4	30 g/L		
$(NH_4)_2SO_4$	20 g/L		
**Solution B-1**			10 ml
$MgSO_4 \cdot 7H_2O$	5 g/L	50 mg/L	
$MnSO_4 \cdot 5H_2O$	0.5418 g/L	5.4 mg/L	
**Solution B-2**			10 ml
$FeSO_4 \cdot 7H_2O$	0.28 g/L	2.8 mg/L	
**Solution C**			25 ml
40% Glucose	40%(w/v)	1%	
**Solution D**			10 ml
Biotin	10 mg/L	0.1 mg/L	
Nicotinic acid	200 mg/L	2 mg/L	
D-Panthothenic acid	200 mg/L	2 mg/L	
Pyridoxyne hydrochloride	400 mg/L	4 mg/L	
Riboflavin	200 mg/L	2 mg/L	
Thiamine hydrochloride	200 mg/L	2 mg/L	
**Adenine Solution**			50 ml
Adenine $\cdot$ $1/2H_2SO_4$	300 mg/L	15mg/L	
**Guanine Solution***			50 ml
Guanine	592 mg/L	30mg/L	
**Trace elements Solution**			10 ml
$CaCl_2$	109.94 mg/L	**	
1M $ZnSO_4$	105.2 ul	0.17mg/L	
100mM $CuSO_4$	504 ul	0.08mg/L	
$CoCl_2 \cdot 6H_2O$	12 mg/L	0.12mg/L	
$Na_2MoO_4 \cdot 2H_2O$	12 mg/L	0.12mg/L	
**Amino Acids Solution *** **			20 ml
**Glutamine Solution**			10 ml
L-Glutamine	29.2 g/L	0.29 g/L	
**Solution Ca**			0.14
$CaCl_2$	55.4987 g/L	8.9mg/L**	

* in 0.05N NaOH

** total

*** RPMI1640 amino acids solution (50X) (Sigma, R7131)

Reuse from Reference: 8, Morikawa et al., 2012

Supplementary References

1. Cafini, F. et al. Horizontal gene transmission of the *cfr* gene to MRSA and *Enterococcus*: role of *Staphylococcus epidermidis* as a reservoir and alternative pathway for the spread of linezolid resistance. *J Antimicrob Chemother* **71**, 587-592, doi:10.1093/jac/dkv391 (2016).

2. Pantůček, R. et al. Identification of bacteriophage types and their carriage in *Staphylococcus aureus*. *Archives of Virology* **149**, 1689–1703 (2004).

3. Wang, L., Safo, M. & Archer, G. L. Characterization of DNA Sequences Required for the CcrAB-Mediated Integration of Staphylococcal Cassette Chromosome *mec*, a *Staphylococcus aureus* Genomic Island. *Journal of Bacteriology* **194**, 486-498, doi:10.1128/Jb.05047-11 (2012).

4. Kreiswirth, B. N. et al. The toxic shock syndrome exotoxin structural gene is not detectably transmitted by a prophage. *Nature* **305**, 709–712 (1983).

5. Kuwahara-Arai, K., Kondo, N., Hori, S., Tateda-Suzuki, E. & Hiramatsu, K. Suppression of methicillin resistance in a *mecA*-containing pre-methicillin-resistant *Staphylococcus aureus* strain is caused by the *mecI*-mediated repression of PBP2' production. *Antimicrobial Agents and Chemotherapy* **40**, 2680–2685 (1996).

6. Ito, T., Katayama, Y. & Hiramatsu, K. Cloning and nucleotide sequence determination of the entire *mec* DNA of pre-methicillin-resistant *Staphylococcus aureus* N315. *Antimicrob. Agents Chemother*. **43**, 1449-1458, (1999).

7. Tsai, M. et al. *Staphylococcus aureus* requires cardiolipin for survival under conditions of high salinity. *BMC Microbiol* **11,** 13, doi:10.1186/1471-2180-11-13 (2011).

8. Morikawa, K. et al. Expression of a cryptic secondary sigma factor gene unveils natural competence for DNA transformation in *Staphylococcus aureus*. *PLoS Pathog* **8,** e1003003, doi:10.1371/journal.ppat.1003003 (2012).

9. Inose, Y. et al. Genetic characterization of the natural SigB variants found in clinical isolates of *Staphylococcus aureus*. *The Journal of General and Applied Microbiology* **52**, 259–271 (2006).

10. Noto, M. J., Kreiswirth, B. N., Monk, A. B. & Archer, G. L. Gene acquisition at the insertion site for SCC*mec*, the genomic island conferring methicillin resistance in *Staphylococcus aureus*. *Journal of Bacteriology* **190**, 1276-1283, doi:10.1128/Jb.01128-07 (2008).

11. Pitkälä A., Salmikivi, L., Bredbacka, P., Myllyniemi, A.-L. & Koskinen, M. T. Comparison of tests for detection of β-Lactamase-producing staphylococci. *Journal of Clinical Microbiology* **45**, 2031–2033 (2007).

12. Tasara, T., Cernela, N. & Stephan, R. Function impairing mutations in *blaZ* and *blaR* genes of penicillin susceptible *Staphylococcus aureus* strains isolated from bovine mastitis. *Schweizer Archiv für Tierheilkunde* **155**, 359–363 (2013).

13. Schwalm, N. D., Verghese, B. & Knabel, S. J. A novel multiplex PCR method for detecting the major clonal complexes of MRSA in nasal isolates from a Pennsylvania hospital. *Journal of microbiological methods* **86**, 379-382, doi:10.1016/j.mimet.2011.05.024 (2011).

14. Yoshida, Y. et al. Bacitracin sensing and resistance in *Staphylococcus aureus*. *FEMS Microbiology Letters* **320**, 33–39 (2011).
