# Supplementary material for: Natural transformation allows transfer of SCCmec-mediated methicillin resistance in Staphylococcus aureus biofilms
Source: Nat Commun. 2022 May 5;13:2477. doi: 10.1038/s41467-022-29877-2 (PMC9072672; doi:10.1038/s41467-022-29877-2)
Supplement: Supplementary file 2 — Reporting Summary [file 41467_2022_29877_MOESM2_ESM.pdf]

nature portfolio

Corresponding author(s): Kazuya Morikawa

Last updated by author(s): Mar 12, 2022

Reporting Summary

Nature Portfolio wishes to improve the reproducibility of the work that we publish. This form provides structure for consistency and transparency in reporting. For further information on Nature Portfolio policies, see our Editorial Policies and the Editorial Policy Checklist.

Statistics

For all statistical analyses, confirm that the following items are present in the figure legend, table legend, main text, or Methods section.

n/a	Confirmed	
☐	☒	The exact sample size (*n*) for each experimental group/condition, given as a discrete number and unit of measurement
☐	☒	A statement on whether measurements were taken from distinct samples or whether the same sample was measured repeatedly
☐	☒	The statistical test(s) used AND whether they are one- or two-sided<br>*Only common tests should be described solely by name; describe more complex techniques in the Methods section.*
☒	☐	A description of all covariates tested
☒	☐	A description of any assumptions or corrections, such as tests of normality and adjustment for multiple comparisons
☐	☒	A full description of the statistical parameters including central tendency (e.g. means) or other basic estimates (e.g. regression coefficient) AND variation (e.g. standard deviation) or associated estimates of uncertainty (e.g. confidence intervals)
☐	☒	For null hypothesis testing, the test statistic (e.g. *F*, *t*, *r*) with confidence intervals, effect sizes, degrees of freedom and *P* value noted<br>*Give P values as exact values whenever suitable.*
☒	☐	For Bayesian analysis, information on the choice of priors and Markov chain Monte Carlo settings
☒	☐	For hierarchical and complex designs, identification of the appropriate level for tests and full reporting of outcomes
☒	☐	Estimates of effect sizes (e.g. Cohen's *d*, Pearson's *r*), indicating how they were calculated

Our web collection on statistics for biologists contains articles on many of the points above.

Software and code

Policy information about availability of computer code

Data collection	No custom algorithm/ software was used to collect data.
Data analysis	DNASTAR Lasergene version 17 software was used to analyze DNA sequences. GraphPad Prism (GraphPad Software, version 8.4.3) was used for statistical analysis.

For manuscripts utilizing custom algorithms or software that are central to the research but not yet described in published literature, software must be made available to editors and reviewers. We strongly encourage code deposition in a community repository (e.g. GitHub). See the Nature Portfolio guidelines for submitting code & software for further information.

Data

Policy information about availability of data

All manuscripts must include a data availability statement. This statement should provide the following information, where applicable:

- Accession codes, unique identifiers, or web links for publicly available datasets
- A description of any restrictions on data availability
- For clinical datasets or third party data, please ensure that the statement adheres to our policy

The genome sequence data generated in this study have been deposited in the NCBI Sequence Read Archive under accession code SRP340166 [https://www.ncbi.nlm.nih.gov/sra/?term=SRP340166]. The S. aureus N315 genome sequence used in this study are available in the NCBI GenBank database under the accession code BA000018 [https://www.ncbi.nlm.nih.gov/nuccore/BA000018]. Other relevant data are provided within the paper and its Supplementary Information. Source data are provided with this paper.

Field-specific reporting

Please select the one below that is the best fit for your research. If you are not sure, read the appropriate sections before making your selection.

- [x] Life sciences
- [ ] Behavioural & social sciences
- [ ] Ecological, evolutionary & environmental sciences

For a reference copy of the document with all sections, see nature.com/documents/nr-reporting-summary-flat.pdf

Life sciences study design

All studies must disclose on these points even when the disclosure is negative.

Sample size	No statistical methods were used to determine sample size. The sample size (n) represents independent experiments and is indicated in the figures or figure legends throughout the manuscript. For statistical analysis, at least n = 3 was used.
Data exclusions	No data were excluded.
Replication	All experiments in Figs. 1a-b; 2a-c, 2f and Supplementary Figs. 1b-c; 2; 3; 4b-c; 7; 10a; 11a; 12a-b; 13b-c. were repeated at least three times independently. All experiments in Figs. 4a-c, 4e-g and Supplementary Figs. 5a-c; 6a-c; 8; 9a-b; 10b; 11b; 12c; 13a; 15a-b; 16a-b; 18a-b. were repeated at least twice independently with similar results.
Randomization	Microscopic images to count %GFP or %dsRed positive cells were taken at random (we randomly fixed the field by using the bright field observation first, and then the GFP or dsRed images were taken by switching to fluorescence mode.) Randomization was not required for other experiments as all the samples from the same experiment were measured in parallel at similar time to avoid batch effects.
Blinding	Blinding is not relevant in this study, because it does not affect the quantitative results (bacterial growth, transformation efficiency, fluorescence intensity, biofilm formation).

Reporting for specific materials, systems and methods

We require information from authors about some types of materials, experimental systems and methods used in many studies. Here, indicate whether each material, system or method listed is relevant to your study. If you are not sure if a list item applies to your research, read the appropriate section before selecting a response.

Materials & experimental systems

n/a	Involved in the study	
x		Antibodies
x		Eukaryotic cell lines
x		Palaeontology and archaeology
x		Animals and other organisms
x		Human research participants
x		Clinical data
x		Dual use research of concern

Methods

n/a	Involved in the study	
x		ChIP-seq
x		Flow cytometry
x		MRI-based neuroimaging
